# User guide for Social Determinants of Health Survey data in the *All of Us* Research Program

**DOI:** 10.1093/jamia/ocae214

**Published:** 2024-08-27

**Authors:** Theresa A Koleck, Caitlin Dreisbach, Chen Zhang, Susan Grayson, Maichou Lor, Zhirui Deng, Alex Conway, Peter D R Higgins, Suzanne Bakken

**Affiliations:** School of Nursing, University of Pittsburgh, Pittsburgh, PA 15261, United States; School of Nursing, University of Rochester, Rochester, NY 14620, United States; Goergen Institute for Data Science, University of Rochester, Rochester, NY 14627, United States; School of Nursing, University of Rochester, Rochester, NY 14620, United States; School of Nursing, University of Pittsburgh, Pittsburgh, PA 15261, United States; School of Nursing, University of Wisconsin-Madison, Madison, WI 53705, United States; School of Nursing, University of Pittsburgh, Pittsburgh, PA 15261, United States; School of Nursing, University of Pittsburgh, Pittsburgh, PA 15261, United States; School of Medicine, University of Michigan, Ann Arbor, MI 48109, United States; School of Nursing, Columbia University, New York, NY 10032, United States; Department of Biomedical Informatics, Columbia University, New York, NY 10032, United States; Data Science Institute, Columbia University, New York, NY 10027, United States

**Keywords:** big data, social determinants of health, electronic data processing, measurement

## Abstract

**Objectives:**

Integration of social determinants of health into health outcomes research will allow researchers to study health inequities. The *All of Us* Research Program has the potential to be a rich source of social determinants of health data. However, user-friendly recommendations for scoring and interpreting the *All of Us* Social Determinants of Health Survey are needed to return value to communities through advancing researcher competencies in use of the *All of Us* Research Hub Researcher Workbench. We created a user guide aimed at providing researchers with an overview of the Social Determinants of Health Survey, recommendations for scoring and interpreting participant responses, and readily executable R and Python functions.

**Target Audience:**

This user guide targets registered users of the *All of Us* Research Hub Researcher Workbench, a cloud-based platform that supports analysis of *All of Us* data, who are currently conducting or planning to conduct analyses using the Social Determinants of Health Survey.

**Scope:**

We introduce 14 constructs evaluated as part of the Social Determinants of Health Survey and summarize construct operationalization. We offer 30 literature-informed recommendations for scoring participant responses and interpreting scores, with multiple options available for 8 of the constructs. Then, we walk through example R and Python functions for relabeling responses and scoring constructs that can be directly implemented in Jupyter Notebook or RStudio within the Researcher Workbench. Full source code is available in supplemental files and GitHub. Finally, we discuss psychometric considerations related to the Social Determinants of Health Survey for researchers.

## Introduction

Health outcomes are influenced by many elements, including biological, behavioral, social, and environmental factors. Social determinants of health are defined as “the conditions in the environment where people are born, learn, work, play, worship, and age.”^1^ Healthy People 2030, a 10-year initiative in the United States that sets objectives to improve health and well-being (health.gov/healthypeople), has categorized social determinants of health into 5 domains: Economic Stability, Education Access and Quality, Health Care Access and Quality, Neighborhood and Built Environment, and Social and Community Context. Social determinants of health are significant contributors to health, well-being, and quality of life as well as health disparities and inequities.^1^ Despite the Healthy People 2030 framework and known impact on health outcomes, measures used to analyze and report the effects of social determinants of health on outcomes have not been standardized.^2^ Integration of social determinants of health into health outcomes research may allow researchers to better address the sources of health inequities.

The *All of Us* Research Program (allofus.nih.gov) provides an unprecedented opportunity to examine biomedical and health research questions using data collected from diverse and historically underrepresented populations living in the United States. The goal of the *All of Us* Research Program is to improve our understanding of health and disease, reducing health disparities.^3^ As of June 2024, *All of Us* has over 810 000 participants enrolled. More than 555 000 participants completed the required initial steps of the program, which include (1) consenting; (2) agreeing to share electronic health records; (3) completing The Basics, Overall Health, and Lifestyle Surveys; (4) providing physical measurements; and (5) donating at least one biospecimen. Of great interest to researchers investigating the combined effects of biomedical and health variables with social and environmental variables, *All of Us* launched its optional 80-item Social Determinants of Health Survey in November 2021 with the initial data release in January 2023. Almost 30% of *All of Us* participants (approximately *n = *117 800) have responses available for the Social Determinants of Health Survey in the most recent data release (ie, version 7; last date of available data—June 30, 2022). While item sources are clearly cited and described within the *All of Us* Research Hub *Survey Explorer* (researchallofus.org/data-tools/survey-explorer/), there is limited information on determinants evaluated and guidance for scoring and interpreting data.

Our user guide returns value to communities in 3 ways. First, explicit recommendations and readily executable code for scoring and interpreting participant responses on the Social Determinants of Health Survey will advance community and academic researcher competencies in use of the *All of Us* Research Hub (researchallofus.org) Researcher Workbench. Second, the user guide facilitates investigation of social determinants of health and, consequently, the advancement of health equity research, through providing instructions on the operationalization, wrangling, scoring, and interpretation of social determinants of health variables from the *All of Us* Research Program Social Determinants of Health Survey. Third, the user guide facilitates standard reporting and analysis and can be used for research and policy generation related to a variety of high-priority health outcomes across health-disparate communities.

## *All of Us* Social Determinants of Health Survey

The *All of Us* Research Program Social Determinants of Health Survey^4^ combines 80 items from multiple instruments to evaluate 14 constructs (ie, theoretical concepts; Table 1). The items evaluate self-reported perceptions of an individual’s social and physical environment rather than structural social determinants of health. For a detailed description of the approach and process used to develop the Social Determinants of Health Survey, information on decisions to include specific instruments and items, and characteristics of *All of Us* Research Program participants who responded to the Social Determinants of Health Survey, we kindly refer the reader to the technical report prepared by Tesfaye et al.^5^ While largely aligned, we would like the reader to note that because our efforts to increase the usability of the Social Determinant of Health Survey occurred prior to, and independently of, the release of the technical report by Tesfaye et al, our interpretation of the constructs and recommended scoring instructions and interpretation may differ from, complement, and/or offer alternatives to those of Tesfaye et al depending on the construct.^5^

**Table 1.** Social determinants of health constructs evaluated within the *All of Us* Social Determinants of Health Survey.

Construct	Definition	Number of items	Instrument references
Neighborhood cohesion	Collective efficacy and relationships among neighbors that bring them together.^6^^,^^7^	4	^6^ ^,^ ^8^
Neighborhood disorder	“Conditions and activities, both major and minor, criminal and non-criminal, that residents perceive to be signs of breakdown of social order.”^9^	13	^9^ ^,^ ^10^
Neighborhood environment	Neighborhood environment built or social attributes include walkability, housing, transportation, recreational facilities, and crime.^11^	8	^11–13^
Social support	“The provision of assistance or comfort to others, typically to help them cope with biological, psychological, and social stressors.”^14^	8	^15^ ^,^ ^16^
Loneliness	Undesirable “feeling of being cutoff or separated from others.”^17^	8	^17^ ^,^ ^18^
Perceived everyday discrimination	“Individuals’ perception of negative attitude, judgment, or unfair treatment due to their specific characteristics such as gender, race, ethnicity, and social status.”^19^	10	^20–24^
Perceived discrimination in healthcare settings	Perception of mistreatment when going to the office of a doctor or other healthcare provider.	7	^25–29^
Food insecurity	Inability to access adequate food due to lack of money or resources.^30^	2	^31^
Housing insecurity/instability	Lack of consistent or secure shelter.^32^	1	^33^ ^,^ ^34^
Housing quality	“Physical conditions of a person’s home as well as the quality of the social and physical environment in which the home is located.”^35^	1	^36^ ^,^ ^37^
Perceived stress	Subjective appraisal of stress related to life events.^38^	10	^38–40^
Daily spiritual experiences	Perception of the transcendent in an individual’s life.	6	^41^ ^,^ ^42^
Religious service attendance	Frequency with which an individual participates in worship, meetings, or other religious activities.	1	^43^
English proficiency	Ability to speak, write, read, and understand the English language.	1	^44^ ^,^ ^45^

*Note.* Constructs are presented in the order of items on the *All of Us* Social Determinants of Health Survey (researchallofus.org/data-tools/survey-explorer/).

**Figure 1.**
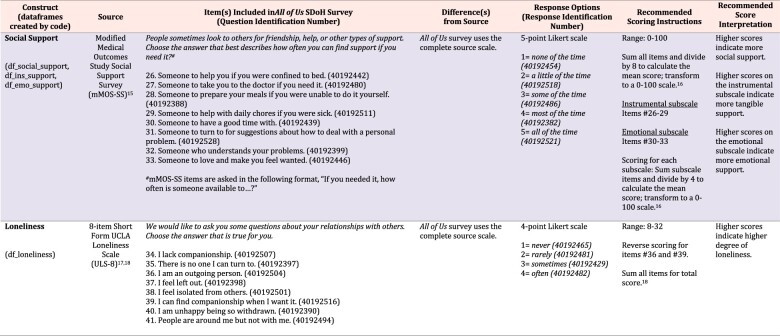
Excerpt of *Supplemental File 1. Representation of Social Determinants of Health Constructs within the All of Us Research Program* for social support and loneliness.

We examined source instruments and searched published literature to summarize the operationalization of each construct within the Social Determinants of Health Survey. While the Social Determinants of Health Survey is available to participants in both English and Spanish, our review of source instruments and published literature was limited to English versions of instruments. For each construct, Supplemental File 1 summarizes the source instrument; item(s) included on the Social Determinants of Health Survey; difference(s) from the source instrument; possible response options; recommended scoring instructions; recommended score interpretation; and name(s) of the dataframe(s) constructed by functions for relabeling and scoring constructs. Specifically, we provide R and Python functions to generate 30 unique dataframes, with multiple scoring options available for 8 of the 14 constructs. See Figure 1 for a preview of Supplemental File 1. The following section provides the conceptual definition of each social determinants of health construct, the operational definition in the source instrument versus the Social Determinants of Health Survey, and recommended scoring instructions and score interpretation. Constructs are presented in the order of items on the Social Determinants of Health Survey (researchallofus.org/data-tools/survey-explorer/).

## Social determinants of health construct descriptions and scoring

### Neighborhood cohesion

Neighborhood cohesion is defined as the collective efficacy and relationships among neighbors that bring them together.^6^^,^^7^ Neighborhood cohesion is measured using a modified 4-item version^8^ of the Social Cohesion Neighborhood Scale.^6^ The source scale is comprised of 5 5-point Likert (*1 = strongly disagree* to *5 = strongly agree*) items. In the Social Determinants of Health Survey, 1 item, “This is a close-knit neighborhood,” is not included. Also, in contrast to the source scale, all items on the Social Determinants of Health Survey are positively stated. Participant responses from the 4 neighborhood cohesion items are summed and divided by 4 to calculate a mean score, ranging from 1 to 5. Higher mean scores are indicative of higher social cohesion within the neighborhood.

### Neighborhood disorder

Neighborhood disorder is defined as the “conditions and activities, both major and minor, criminal and non-criminal, that residents perceive to be signs of breakdown of social order.”^9^ The Social Determinants of Health Survey uses 13 items of the 15-item 4-point Likert (*1 = strongly disagree* to *4 = strongly agree*) Perceived Neighborhood Disorder Scale to measure physical and social neighborhood order and disorder.^9^^,^^10^ The dropped items, “The police protection in my neighborhood is adequate” and “I can trust most people in my neighborhood” assess social order. After reverse scoring order-oriented items to align with disorder-oriented items, participant responses from the 13 items are summed and divided by 13 to calculate a mean score, ranging from 1 to 4. We also provide code to calculate the 6-item physical disorder and order and 7-item social disorder and order subscales.^9^ Higher mean scores indicate neighborhood disorder, while lower mean scores indicate order.

### Neighborhood environment

Neighborhood environment built or social attributes include walkability, housing, transportation, recreational facilities, and crime.^11^ The Social Determinants of Health Survey features 8 items, including all 7 “core items,”^13^ of the 17-item Physical Activity Neighborhood Environment Scale (PANES).^12^ For this scale, a neighborhood is defined as a 10-15 minute walk from an individual’s house.^11^ The first categorical-response item on the PANES evaluates residential density with detached single family housing indicative of low residential density. The remaining items utilize a 4-point Likert (*1 = strongly disagree* to *4 = strongly agree*) scale. After reserve scoring the 2 items related to neighborhood walkability, which are negatively worded (ie, “unsafe”), and eliminating items with responses of *don’t know/not sure* and *does not apply to my neighborhood*, items are summed to generate a total score ranging from 7 to 28. Higher scores indicate greater environmental support for physical activity. While the complete scale is not used, the Social Determinants of Health Survey includes all 6 PANES items used to calculate the Neighborhood Environment Index (NEI).^11^ Higher scores on the NEI, which range from 0 to 6, reflect a more favorable built environment for physical activity.^11^ The additional 2 neighborhood walkability items included on the Social Determinants of Health Survey, that is, “The crime rate in my neighborhood makes it unsafe to go on walks at night” and “The crime rate in neighborhood makes it unsafe to go on walks during the day,” can be used to calculate a crime safety subscale.^13^

### Social support

Using the complete 8-item modified Medical Outcomes Study Social Support Survey (mMOS-SS), the Social Determinants of Health Survey measures social support from family, friends, and community relationships.^15^^,^^46^ Within the mMOS-SS, the 5-point Likert (*1 = none of the time* to *5 = all of the time*) items can be used to calculate an overall score as well as 2 subscales, instrumental support, referring to tangible support (eg, preparing meals and assisting with chores) and emotional support (eg, having someone to have a good time with or help address problems).^15^ The overall scale and subscales are scored by calculating the item average and transforming to a 0 to 100 scale, with higher scores signifying more support.^16^^,^^46^

### Loneliness

Loneliness is defined as the undesirable “feeling of being cutoff or separated from others.”^17^ Within the Social Determinants of Health Survey, loneliness is measured using the complete 8-item Short Form UCLA Loneliness Scale (ULS-8).^17^^,^^18^ ULS-8 items are scored on a 4-point Likert scale from 1 (*never*) to 4 (*always*). After reverse scoring 2 positively worded items (ie, “I am an outgoing person” and “I can find companionship when I want it”), participant responses are summed to calculate a total score, ranging from 8 to 32. Higher scores indicate a higher degree of loneliness.

### Perceived everyday discrimination

Perceived discrimination is defined as “individuals’ perception of negative attitude, judgment, or unfair treatment due to their specific characteristics such as gender, race, ethnicity, and social status.”^19^ The Everyday Discrimination Scale (EDS) “attempts to measure more chronic, routine, and relatively minor experiences of unfair treatment” as opposed to major experiences of discrimination or unfair treatment.^20^^,^^24^ The EDS is comprised of 9 6-point Likert (*1 = never* to *6 = almost every day*) items measuring the frequency of experiencing unfair treatment and one follow-up item evaluating what the respondent believes is the main reason for the experiences, for example, gender, race, religion, or weight. The complete EDS is used to measure perceived everyday discrimination in the Social Determinants of Health Survey. Situation-, frequency-, and chronicity-based scoring are used for the 9 Likert scale items.^21^ For situation-based scoring, participant responses are dichotomized as 0 (*never*) and 1 (*ever*) and summed to calculate a total score (range 0-9). For frequency-based scoring, participant responses are summed to calculate a total score (range 9-54). Frequency-based scoring aims to quantify discrimination experience. For chronicity-based scoring, participant responses are recoded to reflect the number of reported discrimination experiences per year (range 0-2340). For example, the item score for reporting that “you are treated with less respect than other people are” *almost every day* is 260 discrimination experiences per year, calculated as 5 discrimination experiences per week multiplied by 52 weeks per year. Chronicity-based scoring aims to capture intensity and persistence of discrimination experience. Higher scores reflect more frequent perceived experience of unfair treatment.

### Perceived discrimination in healthcare settings

The Discrimination in Medical Settings (DMS) Scale is a modified version of the EDS adapted to the healthcare setting.^25^ The complete 7-item DMS Scale is included in the Social Determinants of Health Survey and features 5-point Likert (*1 = never* to *5 = always*) items that ask participants about the frequency of prior experiences of mistreatment when going to the office of a doctor or other healthcare provider.^25^ Multiple methods to score this scale have been reported in the literature, including never-ever (ie, dichotomize as 2 levels across all items as *none* or *any*)^26^^,^^28^; count scoring (ie, dichotomize each item as 0 [*never*] or 1 [*ever*] and sum [range 0-7])^26–28^; continuous scoring—sum of items (ie, sum items for a total score [range 7-35])^29^; and continuous scoring—item average (ie, sum items and divide by 7 [range 1-5]).^25^^,^^27^ Higher scores indicate greater perceived discrimination in healthcare.

### Food insecurity

A household is considered food insecure if they are unable to access adequate food due to lack of money or resources,^30^ with adequate defined as “quantity or quality of food for all household members to maintain an active lifestyle at all times.”^31^ Food security is measured in the Social Determinants of Health Survey using the 2 items that comprise the Children’s HealthWatch The Hunger Vital Sign: (1) “Within the past 12 months, we worried whether our food would run out before we got money to buy more” and (2) “Within the past 12 months, the food we bought just didn’t last and we didn’t have money to get more.” Items are scored on a scale of *often true*, *sometimes true*, and *never true*, with responses of *often true* or *sometimes true* for either or both items indicating at risk or currently experiencing food insecurity.

### Housing insecurity/instability

Housing insecurity/instability is defined as the lack of consistent or secure shelter.^32^ The Social Determinants of Health Survey measures housing insecurity using 1 of the 3 items from the Health Begins Upstream Risk Screening Tool housing insecurity domain, “In the last 12 months, how many times have you or your family moved from one home to another?”^33^^,^^34^ Two or more moves in the past year indicate at risk or currently experiencing housing insecurity. The 2 items not included in the Social Determinants of Health Survey are as follows: “In the last month, have you slept outside, in a shelter, or in a place not meant for sleeping?” and “In the last month, have you had concerns about the condition or quality of your housing?”

### Housing quality

Housing quality is defined as the “physical conditions of a person’s home as well as the quality of the social and physical environment in which the home is located.”^35^ Housing quality is measured within the Social Determinants of Health Survey using 1 of 2 items used to screen for housing instability from the Accountable Health Communities Health-Related Social Needs Screening Tool, “Think about the place you live. Do you have problems with any of the following?”^36^^,^^37^ The checklist select-all-that-apply response items include *pests such as bugs, ants, or mice*; *mold*; *lead paint or pipes*; *lack of heat*; *oven or stove not working*; s*ome detectors missing or not working*; *water leaks*; or *none of the above*. Participant endorsement of 1 or more items, with the exception of *none of the above*, reflects a housing need. The item from the source survey not included in the Social Determinants of Health Survey is this: “What is your housing situation today?”

### Perceived stress

Perceived stress is a construct representing an individual’s subjective appraisal of stress related to life events.^38^ Perceived stress is measured in the Social Determinants of Health Survey via the 10-item Perceived Stress Scale (PSS-10).^38–40^ PSS-10 items are scored on a 5-point Likert scale (*0 = never* to *4 = very often*). After reverse scoring 4 positively worded items (ie, “how often have you felt confident about your ability to handle your personal problems?”, “how often have you felt that things were going your way?”, “how often have you been able to control irritations in your life?”, and “how often have you felt that you were on top of things?”), responses are summed for a total score, ranging from 0 to 40. When interpreting total scores, higher scores indicate higher levels of perceived stress, with scores of 0-13 categorized as “low stress,” 14-26 categorized as “moderate stress,” and 27-40 categorized as “high stress.”^40^

### Daily spiritual experiences

Items measuring religiousness and spirituality are adapted from the Brief Multidimensional Measure of Religiousness/Spirituality—Daily Spiritual Experiences Scale (DSES) Short Form.^41^^,^^42^ The 6-item DSES is designed to measure an individual’s perception of the transcendent in their life on a 6-point Likert scale (*1 = never or almost never* to *6 = many times a day*).^41^^,^^42^ In the Social Determinants of Health Survey, “(or a higher power)” or “(or a higher power’s)” was added after “God”/”God’s” to 3 items, and 2 response options (ie, *I do not believe in God or a higher power* or *I am not religious*) were added to 4 items. We code these 2 additional response options as equivalent to *1 *=* never or almost never*. Participant responses are summed to generate a total score, ranging from 6 to 36.^41^ Higher total scores indicate more daily religious or spiritual experiences. Tesfaye et al offer an alternative scoring option of setting the added response options of *I do not believe in God or a higher power* or *I am not religious* to equal “0”.^5^ Decisions on how best to treat/collapse participant responses should be informed by the research question.

### Religious service attendance

Religious attendance represents the frequency with which an individual participates in worship, meetings, or other religious activities. Religious attendance is measured within the Social Determinants of Health Survey using a single item from the Nurse’s Health Study, “How often do you go to religious meetings or services?”^43^ The resulting response to this item describes study participant frequency of attending religious meetings or services from *never (or almost never)* to *more than once a week*. In the Social Determinants of Health Survey, one response option, *I am not religious*, was added. We code this additional response option as a unique separate category. Researchers can consider further collapse of categories.

### English proficiency

The Social Determinants of Health Survey evaluates a participant’s primary language used at home and their self-reported proficiency in speaking the English language using 1 item with branching logic adapted from the United States Census, American Community Survey,^44^ and California Health Interview Survey.^45^ The item asks, “Do you speak a language other than English at home?” The branching logic sub-item on proficiency is provided only if the initial response is *yes*. This sub-item states, “Since you speak a language other than English at home, we are interested in your own opinion of how well you speak English. Would you say you speak English…” We provide 3 options for coding English proficiency. The first option dichotomizes whether a participant speaks a language other than English at home as *yes* or *no*. The second option ordinally describes the level of English proficiency for participants who endorsed speaking a language other than English at home as *very well*, *well*, *not well*, *not at all*, *prefer not to answer*, and *don’t know*. The third option further collapses *very well* and *well* into *proficient*, *not well* and *not at all* into *not proficient*, and *prefer not to answer* and *don’t know* into *unknown*. Researchers can consider further collapse or combination of categories.

## R and Python code for scoring social determinants of health constructs

**Figure 2.**
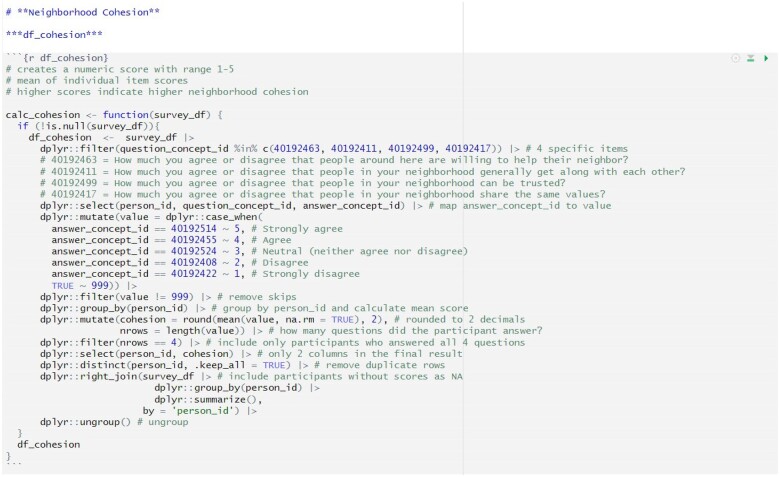
Function to score neighborhood cohesion in R in an RStudio environment.

**Figure 3.**
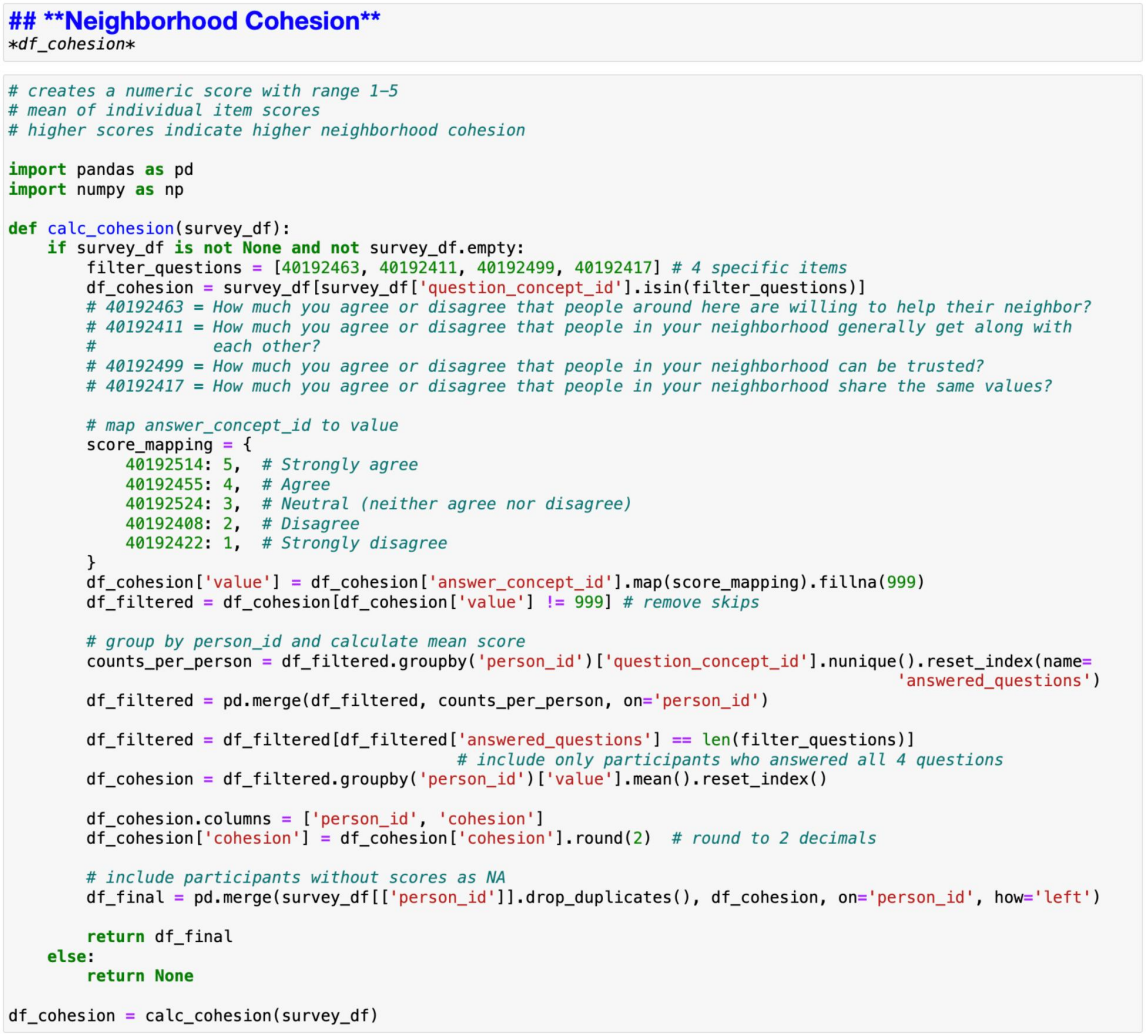
Function to score neighborhood cohesion in Python in a Jupyter Notebook environment.

All analyses of individual-level *All of Us* Research Program data are conducted within the secure, cloud-based Research Hub Researcher Workbench platform. A catalog of active projects in the Researcher Workbench can be accessed at researchallofus.org/research-projects-directory/. After creating a cohort within the Researcher Workbench, registered users can select the “Social Determinants of Health Concept Set” plus any other variables of interest to create a ready-for-analysis dataset. Currently, registered users can use R, Python, or SAS programming languages within the Researcher Workbench. We employed a team approach to code and annotate 30 R and Python functions for relabeling responses and scoring the constructs, with multiple options available for 8 of the 14 constructs, based on the descriptions in the previous section of the user guide. Decisions on the approach to score multiple-item instruments (eg, sum, mean) were based on the method used by the validated source instrument and/or supporting literature. Validation of code accuracy involved checks by multiple team members, calculations from raw data, and comparison of function output between the R and Python code. Recommendations for code and annotation for the neighborhood cohesion function in R in an RStudio environment (Figure 2) and Python in a Jupyter Notebook environment (Figure 3) are provided within the user guide as examples. Source code for all functions is available to researchers through GitHub (github.com/DreisbachLab/AllofUs_SDOH) and supplemental files. Specifically, we provide code in R as an RMarkdown (Supplemental File 2) or Jupyter Notebook (Supplemental File 3) file and in Python as a Jupyter Notebook file (Supplemental File 4). We also provide the functions in R (Supplemental File 5) and Python (Supplemental File 6) in a portable document format.

The functions require the “tidyverse” library for R and the “pandas” and “numpy” libraries for Python and use a “complete case” coding strategy, meaning that only participants who respond to all items required for a calculation will be included in the score calculation. However, participants who do not respond to all items are not discarded from the dataset. Instead, they are temporarily excluded from the specific score calculation and then added back into the dataset with their score marked as “NA” (ie, missing). This approach ensures that all participants remain in the final dataset, even if they have missing responses for some items.

Each function will create a dataframe for the construct or construct scoring option if multiple options exist. For example, *neighborhood cohesion* has one recommended scoring option, a mean score of participant responses to 4 5-point Likert scale items from the Social Cohesion Neighborhood Scale (ie, df_cohesion). In contrast, *perceived everyday discrimination* has 3 scoring options: situation-based (ie, df_edd_situation), frequency-based (ie, df_edd_frequency), and chronicity-based (ie, df_edd_chronicity). The basic steps of the functions are: (1) select the relevant items from the Social Determinants of Health Survey using the unique *All of Us* question_concept_id; (2) map the answer_concept_id to the value; (3) remove skipped items and limit to participants with complete responses; (4) perform calculations; and (5) add back participants without a score as “NA” values in the dataframe. After running the code for a function, researchers simply need to call the function for the construct scoring option that they wish to analyze and enter the name of their dataset, for example, calc_cohension(dataset), to obtain scores.

## Psychometric considerations

We advise researchers to thoughtfully consider the psychometric properties of the items and instruments included within the Social Determinants of Health Survey for their cohort of interest. In the absence of evidence supporting the validity and reliability of an instrument or its use in a new population, researchers should consider the potential influence of the adaptations on interpretations of the resultant scores and comparisons with previous research using the source instrument.

Half of the constructs are evaluated using a complete instrument with evidence of validity and reliability in 1 or more populations (ie, *social support*, mMOS-SS; *loneliness*, ULS-8; *perceived everyday discrimination*, EDS; *perceived discrimination in healthcare settings*, DMS; *perceived stress*, PSS-10; *food insecurity*, The Hunger Vital Sign; and *daily spiritual experiences*, DSES). However, variations in wording from the mMOS-SS and DSES to the Social Determinants of Health Survey were noted for *social support* and *daily spiritual experiences*, respectively. A subset of items from source instruments are used to measure *neighborhood cohesion* (Social Cohesion Neighborhood Scale), *neighborhood disorder* (Perceived Neighborhood Disorder Scale), *neighborhood environment* (PANES), *housing insecurity/instability* (housing insecurity domain of the Upstream Risk Screening Tool), and *housing quality* (housing instability domain of the Accountable Health Communities Health-Related Social Needs Screening Tool) on the Social Determinants of Health Survey. The 2 remaining constructs, *religious service attendance* and *English proficiency*, are measured using single items from larger surveys.

Compared to the source instrument, a response option was added to the item measuring *religious service attendance* (ie, *I am not religious*). Similarly, response options were added to 4 of the 6 items from DSES (ie, *I do not believe in God (or a higher power)* and *I am not religious*). In addition, the Social Determinants of Health Survey has made slight variations to wording in items from the Social Cohesion Neighborhood Scale (*neighborhood cohesion*) and the American Community Survey/California Health Interview Survey (*English proficiency*) as well as the mMOS-SS (*social support*) and DSES (*daily spiritual experiences*) as noted previously.

For multi-item instruments, researchers should report in their manuscript, at a minimum, internal consistency reliability and at least one measure of construct validity.^47^ Internal consistency is an assessment of how well the items in the instrument are related to each other and how consistently they assess the same construct.^48^ Cronbach's alpha is a commonly used coefficient to assess internal consistency reliability. Construct validity is the extent to which an instrument accurately measures the construct it is intended to represent (eg, structural validity through factor analysis).^48^ Internal consistency estimates for the Social Determinants of Health Survey constructs/scales, as conceptualized by Tesfaye and colleagues, demonstrated good to excellent reliability for most scales for participants overall and by demography (ie, underrepresented in biomedical research, racial identity, sex assigned at birth, gender identity, sexual orientation, educational attainment, disability status, and survey language).^5^ While certainly favorable, internal consistency reliability should be conducted for all construct/scale interpretations and in all distinct participant cohorts. Moreover, in interpreting scores, the purpose of the instrument should be considered from multiple perspectives such as individual versus population measurement and diagnostic versus screening purposes.

### Limitations

Our user guide has many strengths that return value to communities through advancing researcher competencies in use of the Researcher Workbench, including comprehensive summarization of social determinants of health construct operationalization, literature-informed recommendations for scoring participant responses and interpreting scores, and transparent source code to facilitate researcher understanding, modification, and customization. We would, however, like to call attention to limitations of the user guide. First, the functions in the user guide employ a complete case coding strategy. We do not address methods for dealing with missing data from multi-item instruments. Fortunately, item non-response on the Social Determinants of Health Survey is low; item non-response, however, has been reported to vary by racial identity, educational attainment, and survey language.^5^ We advise researchers to evaluate missingness and consider strategies for mitigating the influence of missing data and bias related to missingness when implementing the user guide functions. Depending on factors such as the specific research question, participant population, and extent/randomness of missing data, strategies such as casewise deletion, imputation, or scoring based on the subset of items with valid responses^5^ may be employed. Second, we only reviewed English versions of source instruments. The Social Determinants of Health Survey is available to participants in both English and Spanish. Survey administration language has psychometric implications. Finally, recommended scoring instructions and interpretation in this guide are not exhaustive. We remind researchers that decisions on how to select items and score responses collected as part of the Social Determinants of Health Survey should be based on the nature of the individual research question and state of science related to a particular topic and participant population. We invite researchers to use our scoring recommendations and code as a starting point.

## Conclusion

The *All of Us* Research Program dataset represents a massive collective effort to democratize data access to accelerate biomedical and health research. We created a user guide, with accompanying readily executable R and Python functions for response relabeling and scoring, for the Social Determinants of Health Survey to promote researcher use of these constructs. The user guide directly supports community and academic researcher inclusion of social determinants of health in their analyses of *All of Us* data, builds researcher competencies in use of the Researcher Workbench, and provides a foundation for analyses that address high-priority health concerns of communities, including improving health equity, and methods that integrate social determinants of health, electronic health record, and genomic data. It is our hope that specific guidance and functions for scoring and interpreting participant responses will support researcher use of the *All of Us* Social Determinants of Health Survey, thus contributing to the return of value to communities through the advancement of health equity research, increased advocacy for participant groups based on research findings, and policy generation substantiated by *All of Us* researcher findings.

## Supplementary Material

ocae214_Supplementary_Data

## Data Availability

No new data were generated or analyzed in support of this tutorial.
