# Supplementary material for: User guide for Social Determinants of Health Survey data in the All of Us Research Program
Source: J Am Med Inform Assoc. 2024 Aug 27;31(12):3032–41. doi: 10.1093/jamia/ocae214 (PMC11631056; doi:10.1093/jamia/ocae214)
Supplement: ocae214_Supplementary_Data [file ocae214_supplementary_data.zip › ocae214_Supplementary_Data/2024_06_26_Supplemental_File_1_SDoH_Construct_Table.pdf]

Supplemental File 1. Representation of Social Determinants of Health Constructs within the *All of Us* Research Program.

Construct (dataframes created by code)	Source	Item(s) Included in *All of Us* SDoH Survey (Question Identification Number)	Difference(s) from Source	Response Options (Response Identification Number)	Recommended Scoring Instructions	Recommended Score Interpretation
**Neighborhood Cohesion**<br><br>(df_cohesion)	Social Cohesion Neighborhood Scale[6,8]	*The following statements describe what your neighborhood might be like. Tell us how much you agree or disagree.*<br><br>1. People around here are willing to help their neighbors. (40192463)<br>2. People in my[$] neighborhood generally get along with each other. (40192411)<br>3. People in my[$] neighborhood can be trusted. (40192499)<br>4. People in my[$] neighborhood share the same values. (40192417)<br><br>[$]"This" is stated as "my" in the Social Cohesion Neighborhood Scale;[6] "my" is used in the modified 4-item scale.[8]	4 items in modified scale.[8] 5 items in the source scale, 1 additional item: "This is a close-knit neighborhood."[6]<br><br>Items #2 and #4 are negatively stated in the Social Cohesion Neighborhood Scale, i.e., "People in this neighborhood generally do not get along with one another." and "People in this neighborhood do not share the same values." Items #2 and #4 are positively stated in the 4-item modified scale.	5-point Likert scale<br><br>1= *strongly disagree (40192422)*<br>2= *disagree (40192408)*<br>3= *neither agree nor disagree (40192524)*<br>4= *agree (40192455)*<br>5= *strongly agree (40192514)*	Range: 1-5<br><br>Sum all items and divide by 4 to calculate the mean score.	Higher scores indicate higher social cohesion.
**Neighborhood Disorder**<br><br>(df_disorder, df_physical_disorder, df_social_disorder)	Perceived Neighborhood Disorder Scale[9,10]	*The following statements describe what your neighborhood might be like. Tell us how much you agree or disagree.*<br><br>5. There is a lot of graffiti in my neighborhood. (40192420)<br>6. My neighborhood is noisy. (40192522)<br>7. Vandalism is common in my neighborhood. (40192412)<br>8. There are a lot of abandoned buildings in my neighborhood. (40192469)<br>9. My neighborhood is clean. (40192456)<br>10. People in my neighborhood take good care of their houses and apartments. (40192386)<br>11. There are too many people hanging around on the streets near my home. (40192500)<br>12. There is a lot of crime in my neighborhood. (40192493)<br>13. There is too much drug use in my neighborhood. (40192457)<br>14. There is too much alcohol use in my neighborhood. (40192476)<br>15. I'm always having trouble with my neighbors. (40192404)<br>16. In my neighborhood, people watch out for each other. (40192400)<br>17. My neighborhood is safe. (40192384)	15 items in the source scale, 2 additional items: "The police protection in my neighborhood is adequate." and "I can trust most people in my neighborhood."	4-point Likert scale<br><br>1= *strongly disagree (40192422)*<br>2= *disagree (40192408)*<br>3= *agree (40192455)*<br>4 = *strongly agree (40192514)*	Range: 1-4<br><br>Reverse scoring for "order" items #9, #10, #16, and #17.[6]<br><br>Sum all items and divide by 13 to calculate the mean score.<br><br>Physical subscale[9]<br>Items #5-10<br><br>Social subscale[9]<br>Items #11-17<br><br>Scoring for each subscale: Sum subscale items and divide by 6 for physical disorder and 7 for social disorder to calculate the mean score.	Higher scores indicate neighborhood disorder, while lower scores indicate order.

Construct (dataframes created by code)	Source	Item(s) Included in *All of Us* SDoH Survey (Question Identification Number)	Difference(s) from Source	Response Options (Response Identification Number)	Recommended Scoring Instructions	Recommended Score Interpretation
**Neighborhood Environment**<br><br>(df_density, df_spa, df_crime_safety, df_nei)	Physical Activity Neighborhood Environment Scale (PANES)[11-13]	*The next questions ask about what is in your neighborhood. Think about the area around your home that you can walk to in 10–15 minutes.*<br><br>18. What is the main type of housing in your neighborhood? (40192458)<br>19. Many shops, stores, markets or other places to buy things I need are within easy walking distance of my home. (40192436)<br>20. It is within a 10-15 minute walk to a transit stop (such as bus, train, trolley, or tram) from my home. (40192440)<br>21. There are sidewalks on most of the streets in my neighborhood. (40192437)<br>22. There are facilities to bicycle in or near my neighborhood, such as special lanes, separate paths or trails, shared use paths for cycles and pedestrians. (40192431)<br>23. My neighborhood has several free or low-cost recreation facilities, such as parks, walking trails, bike paths, recreation centers, playgrounds, public swimming pools, etc. (4019241)<br>24. The crime rate in my neighborhood makes it unsafe to go on walks at night. (40192414)<br>25. The crime rate in my neighborhood makes it unsafe to go on walks during the day. (40192492)	17 items in the source scale. Items #18-24 are "Core Items," 9 additional recommended or optional items: "There is so much traffic on the streets that it makes it difficult or unpleasant to walk in my neighborhood."; "I see many people being physically active in my neighborhood doing things like walking, jogging, cycling, or playing sports and active games"; "There are many interesting things to look at while walking in my neighborhood."; "How many motor vehicles in working order are there at your household?"; "There are many 4-way intersections in my neighborhood."; "The sidewalks in my neighborhood are well maintained (paved, with few cracks) and not obstructed."; "Places for bicycling (such as bike paths) in and around my neighborhood are well maintained and not obstructed."; "There is so much traffic on the streets that it makes it difficult or unpleasant to ride a bicycle in my neighborhood."; "There are many places to go within easy walking distance of my home."	#18: *detached single-family housing (40192407); townhouses, row house, apartments, or condos of 2-3 stories (40192418); mix of single-family residence and townhouses, row houses, apartments or condos (40192472); apartments or condos of 4-12 stories (40192433); apartments or condos of more than 12 stories (40192409); don't know/not sure (903087)*<br><br>#19-25: 4-point Likert scale<br><br>1= *strongly disagree (40192422)*<br>2= *somewhat disagree (40192527)*<br>3= *somewhat agree (40192478)*<br>4= *strongly agree (40192514)*	<u>Residential density</u>[13]<br>#18: detached single-family housing compared to all others[8]<br><br><u>Support for physical activity (continuous)</u><br>#19-25 Range: 7-28<br><br>Reverse scoring for questions #24 and #25.<br><br>Sum items for total score.<br><br>If respond *don't know/not sure* or *does not apply to my neighborhood*, not used in scored.<br><br><u>Crime safety</u>[13]<br>Range: 1-4<br><br>Sum reverse-scored items #24 and #25 and divide by 2 to calculate the mean score.<br><br><u>Neighborhood Environment Index (NEI)</u>[11,13]<br>Range: 0-6<br><br>Dichotomize #18: 0= single-family housing, 1= other<br><br>Dichotomize #19 through #23: 0= disagree, 1= agree<br><br>Sum of dichotomized #18 through #23 to create index.	<u>Residential density interpretation</u>: Detached single family housing indicates low residential density.<br><br><u>Support for physical activity (continuous) interpretation</u>: Higher total scores indicate greater environmental support for physical activity.<br><br><u>Crime safety interpretation</u>: For questions #24 and #25, higher scores correspond with higher crime safety.<br><br><u>NEI interpretation</u>: Higher scores indicate a more favorable built environment for physical activity.

Construct (dataframes created by code)	Source	Item(s) Included in *All of Us* SDoH Survey (Question Identification Number)	Difference(s) from Source	Response Options (Response Identification Number)	Recommended Scoring Instructions	Recommended Score Interpretation
**Social Support**<br><br>(df_social_support, df_ins_support, df_emo_support)	Modified Medical Outcomes Study Social Support Survey (mMOS-SS)[15]	*People sometimes look to others for friendship, help, or other types of support. Choose the answer that best describes how often you can find support if you need it?#*<br><br>26. Someone to help you if you were confined to bed. (40192442)<br>27. Someone to take you to the doctor if you need it. (40192480)<br>28. Someone to prepare your meals if you were unable to do it yourself. (40192388)<br>29. Someone to help with daily chores if you were sick. (40192511)<br>30. Someone to have a good time with. (40192439)<br>31. Someone to turn to for suggestions about how to deal with a personal problem. (40192528)<br>32. Someone who understands your problems. (40192399)<br>33. Someone to love and make you feel wanted. (40192446)<br><br>#mMOS-SS items are asked in the following format, "If you needed it, how often is someone available to…?"	*All of Us* survey uses the complete source scale.	5-point Likert scale<br><br>1= *none of the time (40192454)*<br>2= *a little of the time (40192518)*<br>3= *some of the time (40192486)*<br>4= *most of the time (40192382)*<br>5= *all of the time (40192521)*	Range: 0-100<br><br>Sum all items and divide by 8 to calculate the mean score; transform to a 0-100 scale.[16]<br><br><u>Instrumental subscale</u><br>Items #26-29<br><br><u>Emotional subscale</u><br>Items #30-33<br><br>Scoring for each subscale: Sum subscale items and divide by 4 to calculate the mean score; transform to a 0-100 scale.[16]	Higher scores indicate more social support.<br><br>Higher scores on the instrumental subscale indicate more tangible support.<br><br>Higher scores on the emotional subscale indicate more emotional support.
**Loneliness**<br><br>(df_loneliness)	8-item Short Form UCLA Loneliness Scale (ULS-8)[17,18]	*We would like to ask you some questions about your relationships with others. Choose the answer that is true for you.*<br><br>34. I lack companionship. (40192507)<br>35. There is no one I can turn to. (40192397)<br>36. I am an outgoing person. (40192504)<br>37. I feel left out. (40192398)<br>38. I feel isolated from others. (40192501)<br>39. I can find companionship when I want it. (40192516)<br>40. I am unhappy being so withdrawn. (40192390)<br>41. People are around me but not with me. (40192494)	*All of Us* survey uses the complete source scale.	4-point Likert scale<br><br>1= *never (40192465)*<br>2= *rarely (40192481)*<br>3= *sometimes (40192429)*<br>4= *often (40192482)*	Range: 8-32<br><br>Reverse scoring for items #36 and #39.<br><br>Sum all items for total score.[18]	Higher scores indicate higher degree of loneliness.

Construct (dataframes created by code)	Source	Item(s) Included in *All of Us* SDoH Survey (Question Identification Number)	Difference(s) from Source	Response Options (Response Identification Number)	Recommended Scoring Instructions	Recommended Score Interpretation
**Perceived Everyday Discrimination**<br><br>(df_edd_situation, df_edd_frequency, df_edd_chronicity)	Everyday Discrimination Scale (EDS)[20-24]	*The next statements describe how others may treat you. In your day-to-day life, how often do any of these happen to you?*<br><br>42. You are treated with less courtesy than other people are. (40192466).<br>43. You are treated with less respect than other people are. (40192489)<br>44. You receive poorer service than other people at restaurants or stores. (40192416)<br>45. People act as if they think you are not smart. (40192490)<br>46. People act as if they are afraid of you. (40192380)<br>47. People act as if they think you are dishonest. (40192395)<br>48. People act as if they're better than you are. (40192496)<br>49. You are called names or insulted. (40192519)<br>50. You are threatened or harassed. (40192451)<br>51. What do you think is the main reason for these experiences? (40192428)	*All of Us* survey uses the complete source scale.	6-point Likert scale<br><br>Questions #42-50<br>1= *never (40192465)*<br>2= *less than once a year (40192464)*<br>3= *a few times a year (40192453)*<br>4= *a few times a month (40192461)*<br>5= *at least once a week (40192391)*<br>6= *almost every day (40192421)*<br><br>Item #51, open ended to evaluate reason for experiences/attribution. *Your ancestry or national origins (40192485), your gender (40192447), your race (40192473), your age (40192427), your religion (40192512), your height (40192450), your weight (40192438), some other aspect of your physical appearance (40192413), your sexual orientation (40192406), your education or income level (40192483), other (specify) (40192467)*	Situation-based scoring[14]<br>Range: 0-9<br>Dichotomize #42-50 as 0= *never*, 1= *ever*; sum items for total score.<br><br>Frequency-based scoring[14]<br>Range: 9-54<br>Sum items for total score.<br><br>Chronicity-based scoring[14]<br>Range: 0-2,340<br>Recode each item to reflect the total number of reported discrimination experiences per year: *never*= 0, *less than once a year*= 0.5x/year (i.e., midpoint 0-1), *a few times a year*= 3x/year (i.e., midpoint 2-4), *a few times a month*= 36x/year (i.e., 3x12 months)l *at least once a week*= 104×/year (i.e., 2x52 weeks), *almost every day*= 260x/year (i.e., 5x52 weeks); Sum for annual total score.<br><br>Can use response to #51 if want to limit attribution to a particular reason (e.g., race).[16]	Higher scores indicate more frequent perceived experience of unfair treatment.

Construct (dataframes created by code)	Source	Item(s) Included in *All of Us* SDoH Survey (Question Identification Number)	Difference(s) from Source	Response Options (Response Identification Number)	Recommended Scoring Instructions	Recommended Score Interpretation
**Perceived Discrimination in Health Care Settings**<br><br>(df_hcd_ever, df_hcd_count, df_hcd_sum, df_hcd_mean)	Discrimination in Medical Settings (DMS) Scale[25-29]	*The next statements describe how others may treat you. How often do any of these happen to you when you go to a doctor's office or other health care provider?*<br><br>52. You are treated with less courtesy than other people. (40192497)<br>53. You are treated with less respect than other people. (40192425)<br>54. You receive poorer service than others. (40192503)<br>55. A doctor or nurse acts [as] if he or she thinks you are not smart. (40192505)<br>56. A doctor or nurse acts as if he or she is afraid of you. (40192423)<br>57. A doctor or nurse acts as if he or she is better than you. (40192383)<br>58. You feel like a doctor or nurse is not listening to what you were saying. (40192394)	*All of Us* survey uses the complete source scale.	5-point Likert scale<br><br>1= *never (40192465)*<br>2= *rarely (40192481)*<br>3= *sometimes (40192429)*<br>4= *most of the time (40192382)*<br>5= *always (40192515)*	Never-ever scoring[26-28]<br>Range: none or any<br><br>Dichotomize as 2 levels across all items as *none* or *any*.<br><br>Count scoring scoring[26-28]<br>Range: 0-7<br><br>Dichotomize each item as 0= *never*, 1= *ever*; sum items for total score.<br><br>Continuous scoring (sum of items) scoring[29]<br>Range: 7-35<br><br>Sum items for total score.<br><br>Continuous scoring (item average)[25,27]<br>Range: 1-5<br><br>Sum items and divide by 7.	Higher scores indicate greater perceived discrimination in health care.
**Food Insecurity**<br><br>(df_food_insecurity)	The Hunger Vital Sign[31]	*The next set of questions asks about food and housing.*<br><br>59. Within the past 12 months, we worried whether our food would run out before we got money to buy more. (40192517)<br>60. Within the past 12 months, the food we bought just didn't last and we didn't have money to get more. (40192426)	*All of Us* survey uses the complete source tool.	*Often true (40192508), sometimes true (40192488), never true (40192474)*	No score is calculated.	Responses of *often true* or *sometimes true* for either or both items indicate at risk or currently experiencing food insecurity.

Construct (dataframes created by code)	Source	Item(s) Included in *All of Us* SDoH Survey (Question Identification Number)	Difference(s) from Source	Response Options (Response Identification Number)	Recommended Scoring Instructions	Recommended Score Interpretation
**Housing Insecurity/ Instability**<br><br>(df_housing_insecurity, df_num_moves)	Upstream Risk Screening Tool[33,34]	61. In the last 12 months, how many times have you or your family moved from one home to another? (40192441)	3 items in the source tool,^ 2 additional questions: In the last month, have you slept outside, in a shelter, or in a place not meant for sleeping?; In the last month, have you had concerns about the condition or quality of your housing?	Continuous open response *0* or more.	No score is calculated.	A response of *2* or more moves in the past year indicates at risk or currently experiencing housing insecurity/ instability.
**Housing Quality**<br><br>(df_housing_quality)	Accountable Health Communities Health-Related Social Needs Screening Tool[36,37]	62.Think about the place you live. Do you have problems with any of the following (check all that apply)? (40192402)	2 items in the source tool,^ one additional question: What is your housing situation today?	Checklist, select all that apply: *Bug infestation (40192460), mold (40192479), lead paint or pipes (40192393), inadequate heat (40192434), oven or stove not working (40192495), no or not working smoke detectors (40192468), water leaks (40192444), none of the above (40192392)*	No score is calculated.	Any positive response indicates a housing need.

Construct (dataframes created by code)	Source	Item(s) Included in *All of Us* SDoH Survey (Question Identification Number)	Difference(s) from Source	Response Options (Response Identification Number)	Recommended Scoring Instructions	Recommended Score Interpretation
**Perceived Stress**<br><br>(df_stress_sum, df_stress_category)	Perceived Stress Scale (PSS-10)[38-40]	*The next questions ask you about your feelings and thoughts during the last month. Please choose how often you felt or thought a certain way.*<br><br>63. In the last month, how often have you been upset because of something that happened unexpectedly? (40192452)<br>64. In the last month, how often have you felt that you were unable to control the important things in your life? (40192381)<br>65. In the last month, how often have you felt nervous and "stressed"? (40192491)<br>66. In the last month, how often have you felt confident about your ability to handle your personal problems? (40192419)<br>67. In the last month, how often have you felt that things were going your way? (40192525)<br>68. In the last month, how often have you found that you could not cope with all the things that you had to do? (40192506)<br>69. In the last month, how often have you been able to control irritations in your life? (40192449)<br>70. In the last month, how often have you felt that you were on top of things? (40192445)<br>71. In the last month, how often have you been angered because of things that happened that were outside of your control? (40192396)<br>72. In the last month, how often have you felt difficulties were piling up so high that you could not overcome them? (40192462)	*All of Us* survey uses the complete source scale.	5-point Likert scale<br><br>0= *never (40192465)*<br>1= *almost never (40192430)*<br>2= *sometimes (40192429)*<br>3= *fairly often (40192477)*<br>4= *very often (40192424)*	Range: 0-40<br><br>Reverse scoring for items #66, #67, #69, and #70.<br><br>Sum responses for the total score.<br><br>Categorical scoring[27]<br>0-13= low stress<br>14-26= moderate stress<br>27-40= high stress	Higher scores indicate higher levels of perceived stress.
**Daily spiritual experiences**<br><br>(df_spirit)	The Brief Multidimensional Measure of Religiousness/ Spirituality – Daily Spiritual Experiences Scale (DSES) Short Form[41,42]	*How often do you experience the following:*<br><br>73. I feel God's (or a higher power's)[#] presence. (40192498)<br>74. I find strength and comfort in my religion. (40192475)<br>75. I feel deep inner peace or harmony. (40192401)<br>76. I desire to be closer to or in union with God (or a higher power).[#] (40192443)<br>77. I feel God's (or a higher power's)[#] love for me, directly or through others. (40192471)<br>78. I am spiritually touched by the beauty of creation. (40192415)<br><br>[#]*All of Us* added "(or a higher power's)" compared to the original scale.	*All of Us* survey uses the complete source scale.	6-point Likert<br><br>1= *never or almost never (40192509), I do not believe in God (or a higher power)[#] (40192487), I am not religious[#] (40192432)*<br>2= *once in a while (40192459)*<br>3= *some days (40192513)*<br>4= *most days (40192484)*<br>5= *everyday (40192385)*<br>6= *many times a day (40192403)*<br><br>[#]*All of Us* added responses to #73, #74, #76, and #77 compared to the source items.	Range: 6-36[41]<br><br>Sum responses for the total score.	Higher scores indicate more daily religious or spiritual experiences.

Construct (dataframes created by code)	Source	Item(s) Included in *All of Us* SDoH Survey (Question Identification Number)	Difference(s) from Source	Response Options (Response Identification Number)	Recommended Scoring Instructions	Recommended Score Interpretation
**Religious Service Attendance**<br><br>(df_religious_attendance)	Nurse Health Study - 2016 Long Version[43]	79. How often do you go to religious meetings or services? (40192470)	49 items in source survey of overall health.^	*More than once a week (706064), once a week (706065), 1 to 3 times per month (706066), less than once per month (706067), never (or almost never) (40192509), #I am not religious (40192432)*<br><br>#*All of Us* added response to #79 compared to the source item.	No score is calculated.	Categorical variable to describe study participants frequency of attending religious meetings or services.
**English Proficiency**<br><br>(df_other_language, df_english_level, df_english_proficient)	United States Census, American Community Survey[44]<br><br>California Health Interview Survey[45]	*The following question asks if you speak any languages other than English at home.*<br><br>80. Do you speak a language other than English at home? (40192526)<br>80a. [If *yes* to the previous question] Since you speak a language other than English at home, we are interested in your own opinion of how well you speak English. Would you say you speak English…(40192529)	2 items in the source survey.^ *All of Us* adapted the wording of the question from: "What languages do you speak at home?"	#80: *Yes (40192448), No (40192523), Prefer not to answer (903079)*<br><br>#80a [if a *Yes* to #80]: *very well (40192435), well (40192510), not well (40192405), not at all (40192387), prefer not to answer (903079), don't know (903087)*	No score is calculated.	Categorical variables to identify participants for whom English is not their primary language and describe proficiency in English

Note. SDoH = Social determinants of health; Superscripts in the "Source" column reference the study/studies that validated the measure. The items in the "Item(s) included in *All of Us* SDoH survey" column are numbered to show ordering of the questions in the *All of Us* Research Program SDoH survey. In the web-based survey, items are not numbered. Superscripts in the "Recommended Scoring Instructions" column provide published papers with scoring standards relevant to the interpretation of the responses. ^= Did not provide all other included items due to length of the measure and the unrelated nature of these items.
