# Supplementary material for: User guide for Social Determinants of Health Survey data in the All of Us Research Program
Source: J Am Med Inform Assoc. 2024 Aug 27;31(12):3032–41. doi: 10.1093/jamia/ocae214 (PMC11631056; doi:10.1093/jamia/ocae214)
Supplement: ocae214_Supplementary_Data [file ocae214_supplementary_data.zip › ocae214_Supplementary_Data/2024_06_26_Supplemental_File_5_R_Functions_PDF.pdf]

All of Us User Guide - R Functions

Load Packages

```r
library(tidyverse)
```

Neighborhood Cohesion

df_cohesion

```
# creates a numeric score with range 1-5
# mean of individual item scores
# higher scores indicate higher neighborhood cohesion

calc_cohesion <- function(survey_df) {
  if (!is.null(survey_df)){
    df_cohesion  <-  survey_df |>
      dplyr::filter(question_concept_id %in% c(40192463, 40192411, 40192499, 40192417)) |> # 4 specific items
      # 40192463 = How much you agree or disagree that people around here are willing to help their neighbor?
      # 40192411 = How much you agree or disagree that people in your neighborhood generally get along with each other?
      # 40192499 = How much you agree or disagree that people in your neighborhood can be trusted?
      # 40192417 = How much you agree or disagree that people in your neighborhood share the same values?
      dplyr::select(person_id, question_concept_id, answer_concept_id) |> # map answer_concept_id to value
      dplyr::mutate(value = dplyr::case_when(
        answer_concept_id == 40192514 ~ 5, # Strongly agree
        answer_concept_id == 40192455 ~ 4, # Agree
        answer_concept_id == 40192524 ~ 3, # Neutral (neither agree nor disagree)
        answer_concept_id == 40192408 ~ 2, # Disagree
        answer_concept_id == 40192422 ~ 1, # Strongly disagree
        TRUE ~ 999)) |>
      dplyr::filter(value != 999) |> # remove skips
      dplyr::group_by(person_id) |> # group by person_id and calculate mean score
      dplyr::mutate(cohesion = round(mean(value, na.rm = TRUE), 2), # rounded to 2 decimals
                    nrows = length(value)) |> # how many questions did the participant answer?
      dplyr::filter(nrows == 4) |> # include only participants who answered all 4 questions
      dplyr::select(person_id, cohesion) |> # only 2 columns in the final result
      dplyr::distinct(person_id, .keep_all = TRUE) |> # remove duplicate rows
      dplyr::right_join(survey_df |> # include participants without scores as NA
                          dplyr::group_by(person_id) |>
                          dplyr::summarize(),
                        by = 'person_id') |>
      dplyr::ungroup() # ungroup
  }
  df_cohesion
}
```

Neighborhood Disorder

Neighborhood Disorder (df_disorder)

```
# creates a numeric score with range 1-4
# mean of individual item scores
# higher scores indicate higher neighborhood disorder, while lower scores indicate order

calc_disorder <- function(survey_df) {
  if (!is.null(survey_df)){
    df_disorder  <-  survey_df |>
    dplyr::filter(question_concept_id %in% c(40192420, 40192522, 40192412, 40192469, 40192456, 40192386, 40192500, 40192493,
                                             40192457, 40192476, 40192404, 40192400, 40192384)) |> # 13 specific items
    # 40192420 = How much you agree or disagree that there is a lot of graffiti in your neighborhood?
    # 40192522 = How much you agree or disagree that your neighborhood is noisy?
    # 40192412 = How much you agree or disagree that vandalism is common in your neighborhood?
    # 40192469 = How much you agree or disagree that there are lot of abandoned buildings in your neighborhood?
    # 40192456 = How much you agree or disagree that your neighborhood is clean?
    # 40192386 = How much you agree or disagree that people in your neighborhood take good care of their houses and
    #            apartments?
    # 40192500 = How much you agree or disagree that there are too many people hanging around on the streets near your home?
    # 40192493 = How much you agree or disagree that there is a lot of crime in your neighborhood?
    # 40192457 = How much you agree or disagree that there is too much drug use in your neighborhood?
    # 40192476 = How much you agree or disagree that there is too much alcohol use in your neighborhood?
    # 40192404 = How much you agree or disagree that you are always having trouble with your neighbors?
    # 40192400 = How much you agree or disagree that in your neighborhood people watch out for each other?
    # 40192384 = How much you agree or disagree that your neighborhood is safe?
    dplyr::select(person_id, question_concept_id, answer_concept_id) |>
      dplyr::mutate(value = dplyr::case_when(
        answer_concept_id == 40192514 ~ 4, # Strongly agree
        answer_concept_id == 40192455 ~ 3, # Agree
        answer_concept_id == 40192408 ~ 2, # Disagree
        answer_concept_id == 40192422 ~ 1, # Strongly disagree
        TRUE ~ 999)) |>
      dplyr::filter(value != 999) |> # remove skips
      dplyr::mutate(value = dplyr::case_when(
        # reverse code for 4 questions
        question_concept_id == 40192456 ~ 5 - value, # 4 -> 1, 3 -> 2, etc
        question_concept_id == 40192386 ~ 5 - value,
```

```
        question_concept_id == 40192400 ~ 5 - value,
        question_concept_id == 40192384 ~ 5 - value,
        TRUE ~ value)) |>
      dplyr::group_by(person_id) |> # group by person_id and calculate mean score
      dplyr::mutate(disorder = round(mean(value,
                                          na.rm = TRUE), 2), # rounded to 2 decimals
                    nrows = length(value)) |> # how many questions did the participant answer?
      dplyr::filter(nrows == 13) |> # include only participants who answered all 13 questions
      dplyr::select(person_id, disorder) |> # only 2 columns in the final result
      dplyr::distinct(person_id, .keep_all = TRUE) |> # remove duplicate rows
      dplyr::right_join(survey_df |> # include participants without scores as NA
                          dplyr::group_by(person_id) |>
                          dplyr::summarize(),
                        by = 'person_id') |>
      dplyr::ungroup() # ungroup
  }
  df_disorder
}
```

Neighborhood Physical Disorder (df__physical__disorder)

```r
# creates a numeric score with range 1-4
# mean of individual item scores
# higher scores indicate higher neighborhood physical disorder, while lower scores indicate physical order

calc_physical_disorder <- function(survey_df) {
  if (!is.null(survey_df)){
    df_physical_disorder  <-  survey_df |>
    dplyr::filter(question_concept_id %in% c(40192420, 40192522, 40192412,
                                             40192469, 40192456, 40192386)) |> # 6 specific items
    # 40192420 = How much you agree or disagree that there is a lot of graffiti in your neighborhood?
    # 40192522 = How much you agree or disagree that your neighborhood is noisy?
    # 40192412 = How much you agree or disagree that vandalism is common in your neighborhood?
    # 40192469 = How much you agree or disagree that there are lot of abandoned buildings in your neighborhood?
    # 40192456 = How much you agree or disagree that your neighborhood is clean?
    # 40192386 = How much you agree or disagree that people in your neighborhood take good care of their houses and
    #            apartments?
    dplyr::select(person_id, question_concept_id, answer_concept_id) |>
      dplyr::mutate(value = dplyr::case_when(
        answer_concept_id == 40192514 ~ 4, # Strongly agree
        answer_concept_id == 40192455 ~ 3, # Agree
        answer_concept_id == 40192408 ~ 2, # Disagree
        answer_concept_id == 40192422 ~ 1, # Strongly disagree
        TRUE ~ 999)) |>
      dplyr::filter(value != 999) |> # remove skips
      dplyr::mutate(value = dplyr::case_when(
        # reverse code for 2 questions
        question_concept_id == 40192456 ~ 5 - value, # 4 -> 1, 3 -> 2, etc
        question_concept_id == 40192386 ~ 5 - value,
        TRUE ~ value)) |>
      dplyr::group_by(person_id) |> # group by person_id and calculate mean score
      dplyr::mutate(physical_disorder = round(mean(value,
                                                   na.rm = TRUE), 2), # rounded to 2 decimals
                    nrows = length(value)) |> # how many questions did the participant answer?
      dplyr::filter(nrows == 6) |> # include only participants who answered all 6 questions
      dplyr::select(person_id, physical_disorder) |> # only 2 columns in the final result
      dplyr::distinct(person_id, .keep_all = TRUE) |> # remove duplicate rows
      dplyr::right_join(survey_df |> # include participants without scores as NA
```

```
                          dplyr::group_by(person_id) |>
                          dplyr::summarize(),
                        by = 'person_id') |>
      dplyr::ungroup() # ungroup
  }
  df_physical_disorder
}
```

Neighborhood Social Disorder (df_social_disorder)

```r
# creates a numeric score with range 1-4
# mean of individual item scores
# higher scores indicate higher neighborhood social disorder, while lower scores indicate social order

calc_social_disorder <- function(survey_df) {
  if (!is.null(survey_df)){
    df_social_disorder  <-  survey_df |>
    dplyr::filter(question_concept_id %in% c(40192500, 40192493, 40192457, 40192476,
                                              40192404, 40192400, 40192384)) |> # 7 specific items
    # 40192500 = How much you agree or disagree that there are too many people hanging around on the streets near your home?
    # 40192493 = How much you agree or disagree that there is a lot of crime in your neighborhood?
    # 40192457 = How much you agree or disagree that there is too much drug use in your neighborhood?
    # 40192476 = How much you agree or disagree that there is too much alcohol use in your neighborhood?
    # 40192404 = How much you agree or disagree that you are always having trouble with your neighbors?
    # 40192400 = How much you agree or disagree that in your neighborhood people watch out for each other?
    # 40192384 = How much you agree or disagree that your neighborhood is safe?
    dplyr::select(person_id, question_concept_id, answer_concept_id) |>
      dplyr::mutate(value = dplyr::case_when(
        answer_concept_id == 40192514 ~ 4, # Strongly agree
        answer_concept_id == 40192455 ~ 3, # Agree
        answer_concept_id == 40192408 ~ 2, # Disagree
        answer_concept_id == 40192422 ~ 1, # Strongly disagree
        TRUE ~ 999)) |>
      dplyr::filter(value != 999) |> # remove skips
      dplyr::mutate(value = dplyr::case_when(
        # reverse code for 2 questions
        question_concept_id == 40192400 ~ 5 - value, # 4 -> 1, 3 -> 2, etc
        question_concept_id == 40192384 ~ 5 - value,
        TRUE ~ value)) |>
      dplyr::group_by(person_id) |> # group by person_id and calculate mean score
      dplyr::mutate(social_disorder = round(mean(value,
                                                  na.rm = TRUE), 2), # rounded to 2 decimals
                    nrows = length(value)) |> # how many questions did the participant answer?
      dplyr::filter(nrows == 7) |> # include only participants who answered all 7 questions
      dplyr::select(person_id, social_disorder) |> # only 2 columns in the final result
      dplyr::distinct(person_id, .keep_all = TRUE) |> # remove duplicate rows
      dplyr::right_join(survey_df |> # include participants without scores as NA
```

```r
                          dplyr::group_by(person_id) |>
                          dplyr::summarize(),
                        by = 'person_id') |>
      dplyr::ungroup() # ungroup
  }
  df_social_disorder
}
```

Neighborhood Environment

Residential Density (df_density)

```
# creates a binary categorical variable with value 'High' or 'Low'
# 'Low' denotes low residential density (i.e., detached single family housing)
# 'High' denotes high residential density

calc_density <- function(survey_df) {
  if (!is.null(survey_df)) {
    df_density <- survey_df |>
      dplyr::filter(question_concept_id == 40192458) |> # 1 specific item
      # 40192458 = What is the main type of housing in your neighborhood?
      dplyr::mutate(density = dplyr::case_when(
        answer_concept_id == 40192407 ~ "Low", # Detached single-family housing
        answer_concept_id %in% c(903087, 903096, 40192520) ~ "none", # Non-answers
        TRUE ~ "High")) |>
      dplyr::filter(density != "none") |> # remove non-answers
      dplyr::select(person_id, density) |>
      dplyr::distinct() # ensure each person_id is represented once

    # Right join with the original survey_df to include participants without scores as NA
    df_density <- survey_df |>
      dplyr::select(person_id) |>
      dplyr::distinct() |> # ensure each person_id is represented once
      dplyr::right_join(df_density, by = "person_id")
  }
  df_density
}
```

Environmental Support for Physical Activity (df_spa)

```r
# creates a numeric score with range 7-28
# sum of individual item scores
# higher scores indicate greater environmental support for physical activity

calc_spa <- function(survey_df) {
  if (!is.null(survey_df)){
    df_spa  <-  survey_df |>
      dplyr::filter(question_concept_id %in% c(40192436, 40192440, 40192437, 40192431, 40192410,
                                               40192492, 40192414)) |> # 7 specific items
      # 40192436 = Many shops, stores, markets or other places to buy things I need are within easy walking distance of
      #            my home. Would you say that you...
      # 40192440 = It is within a 10-15 minute walk to a transit stop (such as bus, train, trolley, or tram) from my home.
      #            Would you say that you...
      # 40192437 = There are sidewalks on most of the streets in my neighborhood. Would you say that you...
      # 40192431 = There are facilities to bicycle in or near my neighborhood, such as special lanes, separate paths or
      #            trails, or shared use paths for cycles and pedestrians. Would you say that you...
      # 40192410 = My neighborhood has several free or low-cost recreation facilities, such as parks, walking trails,
      #            bike paths, recreation centers, playgrounds, public swimming pools, etc. Would you say that you...
      # 40192492 = The crime rate in my neighborhood makes it unsafe to go on walks at night. Would you say that you...
      # 40192414 = The crime rate in my neighborhood makes it unsafe to go on walks during the day. Would you say that you...
      dplyr::select(person_id, question_concept_id, answer_concept_id) |> # map answer_concept_id to value
      dplyr::mutate(value = dplyr::case_when(
        answer_concept_id == 40192514 ~ 4, # Strongly agree
        answer_concept_id == 40192478 ~ 3, # Somewhat agree
        answer_concept_id == 40192527 ~ 2, # Somewhat disagree
        answer_concept_id == 40192422 ~ 1, # Strongly disagree
        TRUE ~ 999)) |>
      dplyr::filter(value != 999) |> # remove skips
      dplyr::mutate(value = dplyr::case_when(
        # reverse code for 2 questions
        question_concept_id == 40192492 ~ 5 - value, # 4 -> 1, 3 -> 2 etc
        question_concept_id == 40192414 ~ 5 - value,
        TRUE ~ value)) |>
      dplyr::group_by(person_id) |> # group by person_id and calculate sum score
      dplyr::mutate(spa = sum(value,
                              na.rm = TRUE),
                    nrows = length(value)) |> # how many questions did the participant answer?
```

```r
      dplyr::filter(nrows == 7) |> # include only participants who answered all 7 questions
      dplyr::select(person_id, spa) |> # only 2 columns in the final result
      dplyr::distinct(person_id, .keep_all = TRUE) |> # remove duplicate rows
      dplyr::right_join(survey_df |> # include participants without scores as NA
                          dplyr::group_by(person_id) |>
                          dplyr::summarize(),
                        by = 'person_id') |>
      dplyr::ungroup() # ungroup
  }
  df_spa
}
```

Crime Safety (df_crime_safety)

```r
# creates a numeric score with range 1-4
# mean of individual item scores
# higher scores indicate higher crime safety

calc_crime_safety <- function(survey_df) {
  if (!is.null(survey_df)){
    df_crime_safety  <-  survey_df |>
      dplyr::filter(question_concept_id %in% c(40192414, 40192492)) |> # 2 specific items
      # 40192414 = The crime rate in my neighborhood makes it unsafe to go on walks during the day. Would you say that you...
      # 40192492 = The crime rate in my neighborhood makes it unsafe to go on walks at night. Would you say that you...
      dplyr::select(person_id, question_concept_id, answer_concept_id) |> # map answer_concept_id to value
      dplyr::mutate(value = dplyr::case_when(
        # reverse code for 2 questions
        answer_concept_id == 40192514 ~ 1, # Strongly agree
        answer_concept_id == 40192478 ~ 2, # Somewhat agree
        answer_concept_id == 40192527 ~ 3, # Somewhat disagree
        answer_concept_id == 40192422 ~ 4, # Strongly disagree
        TRUE ~ 999)) |>
      dplyr::filter(value != 999) |> # remove skips
      dplyr::group_by(person_id) |> # group by person_id and calculate sum score
      dplyr::mutate(crime_safety = round(mean(value,
                                              na.rm = TRUE), 2), # rounded to 2 decimals
                    nrows = length(value)) |> # how many questions did the participant answer?
      dplyr::filter(nrows == 2) |> # include only participants who answered all 2 questions
      dplyr::select(person_id, crime_safety) |> # only 2 columns in the final result
      dplyr::distinct(person_id, .keep_all = TRUE) |> # remove duplicate rows
      dplyr::right_join(survey_df |> # include participants without scores as NA
                          dplyr::group_by(person_id) |>
                          dplyr::summarize(),
                        by = 'person_id') |>
      dplyr::ungroup() # ungroup
  }
  df_crime_safety
}
```

Neighborhood Environment Index (df_nei)

```r
# creates a numeric score with range 0-6
# sum of individual item scores
# higher scores indicate a more favorable built environment for physical activity

calc_nei <- function(survey_df) {
  if (!is.null(survey_df)){
    df_nei  <-  survey_df |>
      dplyr::filter(question_concept_id %in% c(40192410, 40192431, 40192436, 40192437,
                                               40192440, 40192458)) |> # 6 specific items
      # 40192410 = My neighborhood has several free or low-cost recreation facilities, such as parks, walking trails,
      #            bike paths, recreation centers, playgrounds, public swimming pools, etc. Would you say that you...
      # 40192431 = There are facilities to bicycle in or near my neighborhood, such as special lanes, separate paths or
      #            trails, or shared use paths for cycles and pedestrians. Would you say that you...
      # 40192436 = Many shops, stores, markets or other places to buy things I need are within easy walking distance of
      #            my home. Would you say that you...
      # 40192437 = There are sidewalks on most of the streets in my neighborhood. Would you say that you...
      # 40192440 = It is within a 10-15 minute walk to a transit stop (such as bus, train, trolley, or tram) from my home.
      #            Would you say that you...
      # 40192458 = What is the main type of housing in your neighborhood?
      dplyr::select(person_id, question_concept_id, answer_concept_id) |> # map answer_concept_id to value
      dplyr::mutate(value = dplyr::case_when(
        # any legitimate response option other than the first 3 gets coded to a 1
        answer_concept_id == 40192527 ~ 0, # Somewhat disagree
        answer_concept_id == 40192422 ~ 0, # Strongly disagree
        answer_concept_id == 40192407 ~ 0, # Detached single-family housing
        answer_concept_id == 903087 ~ 999, # PMI: Dont Know
        answer_concept_id == 903096 ~ 999, # PMI: Skip
        answer_concept_id == 40192520 ~ 999, # Does not apply to my neighborhood
        TRUE ~ 1)) |>
      dplyr::filter(value != 999) |> # remove skips
      dplyr::group_by(person_id) |> # group by person_id and calculate sum score
      dplyr::mutate(nei = sum(value,
                              na.rm = TRUE),
                    nrows = length(value)) |> # how many questions did the participant answer?
      dplyr::filter(nrows == 6) |> # include only participants who answered all 6 questions
      dplyr::select(person_id, nei) |> # only 2 columns in the final result
      dplyr::distinct(person_id, .keep_all = TRUE) |> # remove duplicate rows
```

```
      dplyr::right_join(survey_df |> # include participants without scores as NA
                          dplyr::group_by(person_id) |>
                          dplyr::summarize(),
                        by = 'person_id') |>
      dplyr::ungroup() # ungroup
  }
  df_nei
}
```

Social Support

Overall Social Support (df_social_support)

```r
# creates a numeric score with range 0-100
# mean of individual item scores transformed to a 0-100 scale
# higher scores indicate more social support

calc_social_support <- function(survey_df) {
  if (!is.null(survey_df)){
    df_social_support  <-  survey_df |>
      dplyr::filter(question_concept_id %in% c(40192388, 40192399, 40192439, 40192442, 40192446,
                                               40192480, 40192511, 40192528)) |> # 8 specific items
      # 40192388 = How often do you have someone to prepare your meals if you were unable to do it yourself?
      # 40192399 = How often do you have someone who understands your problems?
      # 40192439 = How often do you have someone to have a good time with?
      # 40192442 = How often do you have someone to help you if you were confined to bed?
      # 40192446 = How often do you have someone to love and make you feel wanted?
      # 40192480 = How often do you have someone to take you to the doctor if you need it?
      # 40192511 = How often do you have someone to help you with daily chores if you were sick?
      # 40192528 = How often do you have someone to turn to for suggestions about how to deal with a personal problem?
      dplyr::select(person_id, question_concept_id, answer_concept_id) |> # map answer_concept_id to value
      dplyr::mutate(value = dplyr::case_when(
        answer_concept_id == 40192454 ~ 1, # None of the time
        answer_concept_id == 40192518 ~ 2, # A little of the time
        answer_concept_id == 40192486 ~ 3, # Some of the time
        answer_concept_id == 40192382 ~ 4, # Most of the time
        answer_concept_id == 40192521 ~ 5, # All of the time
        TRUE ~ 999)) |>
      dplyr::filter(value != 999) |> # remove skips
      dplyr::group_by(person_id) |> # group by person_id and calculate mean score
      dplyr::mutate(social_support = round(100*(sum(value, na.rm = TRUE)-8)/(40-8),
                                           2), # rounded to 2 decimals
                    nrows = length(value)) |> # how many questions did the participant answer?
      dplyr::filter(nrows == 8) |> # include only participants who answered all 8 questions
      dplyr::select(person_id, social_support) |> # only 2 columns in the final result
      dplyr::distinct(person_id, .keep_all = TRUE) |> # remove duplicate rows
      dplyr::right_join(survey_df |> # include participants without scores as NA
                          dplyr::group_by(person_id) |>
```

```
                          dplyr::summarize(),
                        by = 'person_id') |>
      dplyr::ungroup() # ungroup
  }
  df_social_support
}
```

Instrumental Subscale (df_ins_support)

```r
# creates a numeric score with range 0-100
# mean of individual item scores transformed to a 0-100 scale
# higher scores indicate more tangible support

calc_ins_support <- function(survey_df) {
  if (!is.null(survey_df)){
    df_ins_support  <-  survey_df |>
      dplyr::filter(question_concept_id %in% c(40192388, 40192442, 40192480, 40192511)) |> # 4 specific items
      # 40192388 = How often do you have someone to prepare your meals if you were unable to do it yourself?
      # 40192442 = How often do you have someone to help you if you were confined to bed?
      # 40192480 = How often do you have someone to take you to the doctor if you need it?
      # 40192511 = How often do you have someone to help you with daily chores if you were sick?
      dplyr::select(person_id, question_concept_id, answer_concept_id) |> # map answer_concept_id to value
      dplyr::mutate(value = dplyr::case_when(
        answer_concept_id == 40192454 ~ 1, # None of the time
        answer_concept_id == 40192518 ~ 2, # A little of the time
        answer_concept_id == 40192486 ~ 3, # Some of the time
        answer_concept_id == 40192382 ~ 4, # Most of the time
        answer_concept_id == 40192521 ~ 5, # All of the time
        TRUE ~ 999)) |>
      dplyr::filter(value != 999) |> # remove skips
      dplyr::group_by(person_id) |> # group by person_id and calculate mean score
      dplyr::mutate(ins_support = round(100*(sum(value, na.rm = TRUE)-4)/(20-4),
                                        2), # rounded to 2 decimals
                    nrows = length(value)) |> # how many questions did the participant answer?
      dplyr::filter(nrows == 4) |> # include only participants who answered all 4 questions
      dplyr::select(person_id, ins_support) |> # only 2 columns in the final result
      dplyr::distinct(person_id, .keep_all = TRUE) |> # remove duplicate rows
      dplyr::right_join(survey_df |> # include participants without scores as NA
                          dplyr::group_by(person_id) |>
                          dplyr::summarize(),
                        by = 'person_id') |>
      dplyr::ungroup() # ungroup
  }
  df_ins_support
}
```

Emotional Subscale (df_emo_support)

```r
# creates a numeric score with range 0-100
# mean of individual item scores transformed to a 0-100 scale
# higher scores indicate more emotional support

calc_emo_support <- function(survey_df) {
  if (!is.null(survey_df)){
    df_emo_support  <-  survey_df |>
      dplyr::filter(question_concept_id %in% c(40192399, 40192439, 40192446, 40192528)) |> # 4 specific items
      # 40192399 = How often do you have someone who understands your problems?
      # 40192439 = How often do you have someone to have a good time with?
      # 40192446 = How often do you have someone to love and make you feel wanted?
      # 40192528 = How often do you have someone to turn to for suggestions about how to deal with a personal problem?
      dplyr::select(person_id, question_concept_id, answer_concept_id) |> # map answer_concept_id to value
      dplyr::mutate(value = dplyr::case_when(
        answer_concept_id == 40192454 ~ 1, # None of the time
        answer_concept_id == 40192518 ~ 2, # A little of the time
        answer_concept_id == 40192486 ~ 3, # Some of the time
        answer_concept_id == 40192382 ~ 4, # Most of the time
        answer_concept_id == 40192521 ~ 5, # All of the time
        TRUE ~ 999)) |>
      dplyr::filter(value != 999) |> # remove skips
      dplyr::group_by(person_id) |> # group by person_id and calculate mean score
      dplyr::mutate(emo_support = round(100*(sum(value, na.rm = TRUE)-4)/(20-4),
                                        2), # rounded to 2 decimals
                    nrows = length(value)) |> # how many questions did the participant answer?
      dplyr::filter(nrows == 4) |> # include only participants who answered all 4 questions
      dplyr::select(person_id, emo_support) |> # only 2 columns in the final result
      dplyr::distinct(person_id, .keep_all = TRUE) |> # remove duplicate rows
      dplyr::right_join(survey_df |> # include participants without scores as NA
                          dplyr::group_by(person_id) |>
                          dplyr::summarize(),
                        by = 'person_id') |>
      dplyr::ungroup() # ungroup
  }
  df_emo_support
}
```

Loneliness

df_loneliness

```
# creates a numeric score with range 8-32
# sum of individual item scores
# higher scores indicate higher degree of loneliness

calc_loneliness <- function(survey_df) {
  if (!is.null(survey_df)){
    df_loneliness  <-  survey_df |>
      dplyr::filter(question_concept_id %in% c(40192390, 40192397, 40192398, 40192494, 40192501,
                                               40192504, 40192507, 40192516)) |> # 8 specific items
      # 40192390 = How often do you feel that you are unhappy being so withdrawn?
      # 40192397 = How often do you feel that there is no one you can turn to?
      # 40192398 = How often do you feel left out?
      # 40192494 = How often do you feel that people are around you but not with you?
      # 40192501 = How often do you feel isolated from others?
      # 40192504 = How often do you feel that you are an outgoing person?
      # 40192507 = How often do you feel lack companionship?
      # 40192516 = How often do you fell that you can find companionship when you want it?
      dplyr::select(person_id, question_concept_id, answer_concept_id) |> # map answer_concept_id to value
      dplyr::mutate(value = dplyr::case_when(
        answer_concept_id == 40192465 ~ 1, # Never
        answer_concept_id == 40192481 ~ 2, # Rarely
        answer_concept_id == 40192429 ~ 3, # Sometimes
        answer_concept_id == 40192482 ~ 4, # Often
        TRUE ~ 999)) |>
      dplyr::filter(value != 999) |> # remove skips
      dplyr::mutate(value = dplyr::case_when(
        # reverse code for 4 questions
        question_concept_id == 40192504 ~ 5 - value, # 4 -> 1, 3 -> 2 etc
        question_concept_id == 40192516 ~ 5 - value,
        TRUE ~ value)) |>
      dplyr::group_by(person_id) |> # group by person_id and calculate sum score
      dplyr::mutate(loneliness = sum(value,
                                     na.rm = TRUE),
                    nrows = length(value)) |> # how many questions did the participant answer?
      dplyr::filter(nrows == 8) |> # include only participants who answered all 8 questions
```

```
    dplyr::select(person_id, loneliness) |> # only 2 columns in the final result
    dplyr::distinct(person_id, .keep_all = TRUE) |> # remove duplicate rows
    dplyr::right_join(survey_df |> # include participants without scores as NA
                        dplyr::group_by(person_id) |>
                        dplyr::summarize(),
                      by = 'person_id') |>
    dplyr::ungroup() # ungroup
  }
  df_loneliness
}
```

Perceived Everyday Discrimination

Situation-based Scoring (df_edd_situation)

```r
# creates a numeric score with range 0-9
# indicates how many questions the participant responded to with something other than 'Never'
# higher scores indicate more frequent perceived experience of unfair treatment

# reason is an optional argument
# can limit to participants who provided a particular reason for discrimination, e.g. race or age
# see survey for options

calc_edd_situation <- function(survey_df, reason) {
  if (!is.null(survey_df)){
    df_edd_situation  <-  survey_df |>
      dplyr::filter(question_concept_id %in% c(40192380, 40192395, 40192416, 40192451, 40192466, 40192489, 40192490,
                                               40192496, 40192519)) |> # 9 specific items
      # 40192380 = In your day-to-day life, how often do people act as if they are afraid of you?
      # 40192395 = In your day-to-day life, how often do people act as if they think you are dishonest?
      # 40192416 = In your day-to-day life, how often do you receive poorer service than other people at restaurants or
      #            stores?
      # 40192451 = In your day-to-day life, how often are you threatened or harassed?
      # 40192466 = In your day-to-day life, how often are you treated with less courtesy than other people?
      # 40192489 = In your day-to-day life, how often are you treated with less respect than other people?
      # 40192490 = In your day-to-day life, how often do people act as if they think you are not smart?
      # 40192496 = In your day-to-day life, how often do people act as if they're better than you are?
      # 40192519 = In your day-to-day life, how often are you called names or insulted?
      dplyr::select(person_id, question_concept_id, answer_concept_id) |> # map answer_concept_id to value
      dplyr::mutate(value = dplyr::case_when(
        answer_concept_id == 40192465 ~ 0, # Never
        answer_concept_id == 903096 ~ 999, # PMI: Skip
        TRUE ~ 1)) |> # any response besides "Never" is treated as 1
      dplyr::filter(value != 999) |> # remove skips
      dplyr::group_by(person_id) |> # group by person_id and calculate sum score
      dplyr::mutate(edd_situation = sum(value,
                                        na.rm = TRUE),
                    nrows = length(value)) |> # how many questions did the participant answer?
      dplyr::filter(nrows == 9) |> # include only participants who answered all 9 questions
      dplyr::select(person_id, edd_situation) |> # only 2 columns in the final result
```

```r
      dplyr::distinct(person_id, .keep_all = TRUE) |> # remove duplicate rows
      dplyr::ungroup() # ungroup
    if (!missing(reason)) { # if reason for perceived discrimination is provided
      x <- survey_df |>
        dplyr::filter(question_concept_id == 40192428 & answer == reason)
      # 40192428 = Discrimination: What do you think is the main reason for these experiences?
      y <- x$person_id # which participants indicated the given reason for discrimination
      df_edd_situation <- df_edd_situation |>
        dplyr::filter(person_id %in% y) # filter to these participants
    }
    df_edd_situation <- df_edd_situation |>
      dplyr::right_join(survey_df |> # include participants without scores as NA
                          dplyr::group_by(person_id) |>
                          dplyr::summarize(),
                        by = 'person_id')
  }
  df_edd_situation
}
```

Frequency-based Scoring (df_edd_frequency)

```
# creates a numeric score with range 9-54
# sum of individual item scores
# higher scores indicate more frequent perceived experience of unfair treatment

# reason is an optional argument
# can limit to participants who provided a particular reason for discrimination, e.g. race or age
# see survey for options

calc_edd_frequency <- function(survey_df, reason) {
  if (!is.null(survey_df)){
    df_edd_frequency  <-  survey_df |>
      dplyr::filter(question_concept_id %in% c(40192380, 40192395, 40192416, 40192451, 40192466, 40192489, 40192490,
                                               40192496, 40192519)) |> # 9 specific items
      # 40192380 = In your day-to-day life, how often do people act as if they are afraid of you?
      # 40192395 = In your day-to-day life, how often do people act as if they think you are dishonest?
      # 40192416 = In your day-to-day life, how often do you receive poorer service than other people at restaurants or
      #            stores?
      # 40192451 = In your day-to-day life, how often are you threatened or harassed?
      # 40192466 = In your day-to-day life, how often are you treated with less courtesy than other people?
      # 40192489 = In your day-to-day life, how often are you treated with less respect than other people?
      # 40192490 = In your day-to-day life, how often do people act as if they think you are not smart?
      # 40192496 = In your day-to-day life, how often do people act as if they're better than you are?
      # 40192519 = In your day-to-day life, how often are you called names or insulted?
      dplyr::select(person_id, question_concept_id, answer_concept_id) |> # map answer_concept_id to value
      dplyr::mutate(value = dplyr::case_when(
        answer_concept_id == 40192465 ~ 1, # Never
        answer_concept_id == 40192464 ~ 2, # Less than once a year
        answer_concept_id == 40192453 ~ 3, # A few times a year
        answer_concept_id == 40192461 ~ 4, # A few times a month
        answer_concept_id == 40192391 ~ 5, # At least once a week
        answer_concept_id == 40192421 ~ 6, # Almost everyday
        TRUE ~ 999)) |>
      dplyr::filter(value != 999) |> # remove skips
      dplyr::group_by(person_id) |> # group by person_id and calculate sum score
      dplyr::mutate(edd_frequency = sum(value,
                                        na.rm = TRUE),
                    nrows = length(value)) |> # how many questions did the participant answer?
```

```r
      dplyr::filter(nrows == 9) |> # include only participants who answered all 9 questions
      dplyr::select(person_id, edd_frequency) |> # only 2 columns in the final result
      dplyr::distinct(person_id, .keep_all = TRUE) |> # remove duplicate rows
      dplyr::ungroup() # ungroup
    if (!missing(reason)) { # if reason for perceived discrimination is provided
      x <- survey_df |>
        dplyr::filter(question_concept_id == 40192428 & answer == reason)
      # 40192428 = Discrimination: What do you think is the main reason for these experiences?
      y <- x$person_id # which participants indicated the given reason for discrimination
      df_edd_frequency <- df_edd_frequency |>
        dplyr::filter(person_id %in% y) # filter to these participants
    }
    df_edd_frequency <- df_edd_frequency |>
      dplyr::right_join(survey_df |> # include participants without scores as NA
                          dplyr::group_by(person_id) |>
                          dplyr::summarize(),
                        by = 'person_id')
  }
  df_edd_frequency
}
```

Chronicity-based Scoring (df_edd_chronicity)

```
# creates a numeric score with range 0-2340
# indicates the total number of discrimination experiences in a year
# higher scores indicate more frequent perceived experience of unfair treatment

# reason is an optional argument
# can limit to participants who provided a particular reason for discrimination, e.g. race or age
# see survey for options

calc_edd_chronicity <- function(survey_df, reason) {
  if (!is.null(survey_df)){
    df_edd_chronicity  <-  survey_df |>
      dplyr::filter(question_concept_id %in% c(40192380, 40192395, 40192416, 40192451, 40192466, 40192489, 40192490,
                                               40192496, 40192519)) |> #9 specific items
      # 40192380 = In your day-to-day life, how often do people act as if they are afraid of you?
      # 40192395 = In your day-to-day life, how often do people act as if they think you are dishonest?
      # 40192416 = In your day-to-day life, how often do you receive poorer service than other people at restaurants or
      #            stores?
      # 40192451 = In your day-to-day life, how often are you threatened or harassed?
      # 40192466 = In your day-to-day life, how often are you treated with less courtesy than other people?
      # 40192489 = In your day-to-day life, how often are you treated with less respect than other people?
      # 40192490 = In your day-to-day life, how often do people act as if they think you are not smart?
      # 40192496 = In your day-to-day life, how often do people act as if they're better than you are?
      # 40192519 = In your day-to-day life, how often are you called names or insulted?
      dplyr::select(person_id, question_concept_id, answer_concept_id) |> # map answer_concept_id to value
      dplyr::mutate(value = dplyr::case_when(
        answer_concept_id == 40192465 ~ 0, # Never
        answer_concept_id == 40192464 ~ 0.5, # Less than once a year
        answer_concept_id == 40192453 ~ 3, # A few times a year
        answer_concept_id == 40192461 ~ 36, # A few times a month
        answer_concept_id == 40192391 ~ 104, # At least once a week
        answer_concept_id == 40192421 ~ 260, # Almost everyday
        TRUE ~ 999)) |>
      dplyr::filter(value != 999) |> # remove skips
      dplyr::group_by(person_id) |> # group by person_id and calculate sum score
      dplyr::mutate(edd_chronicity = sum(value,
                                         na.rm = TRUE),
                    nrows = length(value)) |> # how many questions did the participant answer?
```

```r
      dplyr::filter(nrows == 9) |> # include only participants who answered all 9 questions
      dplyr::select(person_id, edd_chronicity) |> # only 2 columns in the final result
      dplyr::distinct(person_id, .keep_all = TRUE) |> # remove duplicate rows
      dplyr::ungroup() # ungroup
    if (!missing(reason)) { # if reason for perceived discrimination is provided
      x <- survey_df |>
        dplyr::filter(question_concept_id == 40192428 & answer == reason)
      # 40192428 = Discrimination: What do you think is the main reason for these experiences?
      y <- x$person_id # which participants indicated the given reason for discrimination
      df_edd_chronicity <- df_edd_chronicity |>
        dplyr::filter(person_id %in% y) # filter to these participants
    }
    df_edd_chronicity <- df_edd_chronicity |>
      dplyr::right_join(survey_df |> # include participants without scores as NA
                          dplyr::group_by(person_id) |>
                          dplyr::summarize(),
                        by = 'person_id')
  }
  df_edd_chronicity
}
```

Perceived Discrimination in Health Care Settings

Never/Ever Scoring (df_hcd_ever)

```
# creates a binary categorical variable with value TRUE or FALSE
# TRUE denotes that the participant has endorsed perceived discrimination in health care ever

calc_hcd_ever <- function(survey_df) {
  if (!is.null(survey_df)){
    df_hcd_ever  <-  survey_df |>
      dplyr::filter(question_concept_id %in% c(40192383, 40192394, 40192423, 40192425, 40192497,
                                                40192503, 40192505)) |> # 7 specific items
      # 40192383 = How often does a doctor or nurse act as if he or she is better than you when you go to a doctor's office
      #             or other health care provider?
      # 40192394 = How often do you feel like a doctor or nurse is not listening to what you were saying,
      #             when you go to a doctor's office or other health care provider?
      # 40192423 = How often does a doctor or nurse act as if he or she is afraid of you when you go to a doctor's office
      #             or other health care provider?
      # 40192425 = How often are you treated with less respect than other people when you go to a doctor's office
      #             or other health care provider?
      # 40192497 = How often are you treated with less courtesy than other people when you go to a doctor's office
      #             or other health care provider?
      # 40192503 = How often do you receive poorer service than others when you go to a doctor's office
      #             or other health care provider?
      # 40192505 = How often does a doctor or nurse act as if he or she thinks you are not smart when you go to a doctor's
      #             office or other health care provider?
      dplyr::select(person_id, question_concept_id, answer_concept_id) |> # map answer_concept_id to value
      dplyr::mutate(value = dplyr::case_when(
        answer_concept_id == 40192465 ~ 0, # Never
        answer_concept_id == 903096 ~ 999, # PMI: Skip
        TRUE ~ 1)) |> # any response besides "Never" is treated as 1
      dplyr::filter(value != 999) |> # remove skips
      dplyr::group_by(person_id) |> # group by person_id and calculate score
      dplyr::mutate(hcd_ever = 1 %in% value,
                    nrows = length(value)) |> # how many questions did the participant answer?
      dplyr::filter(nrows == 7 | hcd_ever == TRUE) |>
                           # include only participants who answered all 7 questions OR there is a positive
      dplyr::select(person_id, hcd_ever) |> # only 2 columns in the final result
      dplyr::distinct(person_id, .keep_all = TRUE) |> # remove duplicate rows
```

```
      dplyr::right_join(survey_df |> # include participants without scores as NA
                          dplyr::group_by(person_id) |>
                          dplyr::summarize(),
                        by = 'person_id') |>
      dplyr::ungroup() # ungroup
  }
  df_hcd_ever
}
```

Count Scoring (df_hcd_count)

```
# creates a numeric score with range 0-7
# indicates how many items for which the participant endorsed perceived discrimination in health care
# higher scores indicate greater perceived discrimination in health care

calc_hcd_count <- function(survey_df) {
  if (!is.null(survey_df)){
    df_hcd_count  <-  survey_df |>
      dplyr::filter(question_concept_id %in% c(40192383, 40192394, 40192423, 40192425, 40192497,
                                                40192503, 40192505)) |> # 7 specific items
      # 40192383 = How often does a doctor or nurse act as if he or she is better than you when you go to a doctor's office
      #            or other health care provider?
      # 40192394 = How often do you feel like a doctor or nurse is not listening to what you were saying,
      #            when you go to a doctor's office or other health care provider?
      # 40192423 = How often does a doctor or nurse act as if he or she is afraid of you when you go to a doctor's office
      #            or other health care provider?
      # 40192425 = How often are you treated with less respect than other people when you go to a doctor's office
      #            or other health care provider?
      # 40192497 = How often are you treated with less courtesy than other people when you go to a doctor's office
      #            or other health care provider?
      # 40192503 = How often do you receive poorer service than others when you go to a doctor's office
      #            or other health care provider?
      # 40192505 = How often does a doctor or nurse act as if he or she thinks you are not smart when you go to a doctor's
      #            office or other health care provider?
      dplyr::select(person_id, question_concept_id, answer_concept_id) |> # map answer_concept_id to value
      dplyr::mutate(value = dplyr::case_when(
        answer_concept_id == 40192465 ~ 0, # Never
        answer_concept_id == 903096 ~ 999, # PMI: Skip
        TRUE ~ 1)) |> # any response besides "Never" is treated as 1
      dplyr::filter(value != 999) |> # remove skips
      dplyr::group_by(person_id) |> # group by person_id and calculate sum score
      dplyr::mutate(hcd_count = sum(value),
                    nrows = length(value)) |> # how many questions did the participant answer?
      dplyr::filter(nrows == 7) |> # include only participants who answered all 7 questions
      dplyr::select(person_id, hcd_count) |> # only 2 columns in the final result
      dplyr::distinct(person_id, .keep_all = TRUE) |> # remove duplicate rows
      dplyr::right_join(survey_df |> # include participants without scores as NA
                          dplyr::group_by(person_id) |>
```

```
                          dplyr::summarize(),
                        by = 'person_id') |>
      dplyr::ungroup() # ungroup
  }
  df_hcd_count
}
```

Continuous Scoring: Sum of Items (df_hcd_sum)

```r
# creates a numeric score with range 7-35
# sum of individual item scores
# higher scores indicate greater perceived discrimination in health care

calc_hcd_sum <- function(survey_df) {
  if (!is.null(survey_df)){
    df_hcd_sum  <-  survey_df |>
      dplyr::filter(question_concept_id %in% c(40192383, 40192394, 40192423, 40192425, 40192497,
                                               40192503, 40192505)) |> # 7 specific items
      # 40192383 = How often does a doctor or nurse act as if he or she is better than you when you go to a doctor's office
      #            or other health care provider?
      # 40192394 = How often do you feel like a doctor or nurse is not listening to what you were saying,
      #            when you go to a doctor's office or other health care provider?
      # 40192423 = How often does a doctor or nurse act as if he or she is afraid of you when you go to a doctor's office
      #            or other health care provider?
      # 40192425 = How often are you treated with less respect than other people when you go to a doctor's office
      #            or other health care provider?
      # 40192497 = How often are you treated with less courtesy than other people when you go to a doctor's office
      #            or other health care provider?
      # 40192503 = How often do you receive poorer service than others when you go to a doctor's office
      #            or other health care provider?
      # 40192505 = How often does a doctor or nurse act as if he or she thinks you are not smart when you go to a doctor's
      #            office or other health care provider?
      dplyr::select(person_id, question_concept_id, answer_concept_id) |> # map answer_concept_id to value
      dplyr::mutate(value = dplyr::case_when(
        answer_concept_id == 40192465 ~ 1, # Never
        answer_concept_id == 40192481 ~ 2, # Rarely
        answer_concept_id == 40192429 ~ 3, # Sometimes
        answer_concept_id == 40192382 ~ 4, # Most of the time
        answer_concept_id == 40192515 ~ 5, # Always
        TRUE ~ 999)) |>
      dplyr::filter(value != 999) |> # remove skips
      dplyr::group_by(person_id) |> # group by person_id and calculate sum score
      dplyr::mutate(hcd_sum = sum(value),
                    nrows = length(value)) |> # how many questions did the participant answer?
      dplyr::filter(nrows == 7) |> # include only participants who answered all 7 questions
      dplyr::select(person_id, hcd_sum) |> # only 2 columns in the final result
```

```
      dplyr::distinct(person_id, .keep_all = TRUE) |> # remove duplicate rows
      dplyr::right_join(survey_df |> # include participants without scores as NA
                          dplyr::group_by(person_id) |>
                          dplyr::summarize(),
                        by = 'person_id') |>
      dplyr::ungroup() # ungroup
  }
  df_hcd_sum
}
```

Continuous Scoring: Item Average (df_hcd_mean)

```
# creates a numeric score with range 1-5
# mean of individual item scores
# higher scores indicate greater perceived discrimination in health care

calc_hcd_mean <- function(survey_df) {
  if (!is.null(survey_df)){
    df_hcd_mean  <-  survey_df |>
      dplyr::filter(question_concept_id %in% c(40192383, 40192394, 40192423, 40192425, 40192497,
                                               40192503, 40192505)) |> # 7 specific items
      # 40192383 = How often does a doctor or nurse act as if he or she is better than you when you go to a doctor's office
      #            or other health care provider?
      # 40192394 = How often do you feel like a doctor or nurse is not listening to what you were saying,
      #            when you go to a doctor's office or other health care provider?
      # 40192423 = How often does a doctor or nurse act as if he or she is afraid of you when you go to a doctor's office
      #            or other health care provider?
      # 40192425 = How often are you treated with less respect than other people when you go to a doctor's office
      #            or other health care provider?
      # 40192497 = How often are you treated with less courtesy than other people when you go to a doctor's office
      #            or other health care provider?
      # 40192503 = How often do you receive poorer service than others when you go to a doctor's office
      #            or other health care provider?
      # 40192505 = How often does a doctor or nurse act as if he or she thinks you are not smart when you go to a doctor's
      #            office or other health care provider?
      dplyr::select(person_id, question_concept_id, answer_concept_id) |> # map answer_concept_id to value
      dplyr::mutate(value = dplyr::case_when(
        answer_concept_id == 40192465 ~ 1, # Never
        answer_concept_id == 40192481 ~ 2, # Rarely
        answer_concept_id == 40192429 ~ 3, # Sometimes
        answer_concept_id == 40192382 ~ 4, # Most of the time
        answer_concept_id == 40192515 ~ 5, # Always
        TRUE ~ 999)) |>
      dplyr::filter(value != 999) |> # remove skips
      dplyr::group_by(person_id) |> # group by person_id and calculate mean score
      dplyr::mutate(hcd_mean = round(mean(value,
                                          na.rm = TRUE), 2), # rounded to 2 decimals
                    nrows = length(value)) |> # how many questions did the participant answer?
      dplyr::filter(nrows == 7) |> # include only participants who answered all 7 questions
```

```
      dplyr::select(person_id, hcd_mean) |> # only 2 columns in the final result
      dplyr::distinct(person_id, .keep_all = TRUE) |> # remove duplicate rows
      dplyr::right_join(survey_df |> # include participants without scores as NA
                          dplyr::group_by(person_id) |>
                          dplyr::summarize(),
                        by = 'person_id') |>
      dplyr::ungroup() # ungroup
  }
  df_hcd_mean
}
```

Food Insecurity

df_food_insecurity

```
# creates a binary categorical variable with value TRUE or FALSE
# TRUE denotes that the participant is at risk or currently experiencing food insecurity

calc_food_insecurity <- function(survey_df) {
  if (!is.null(survey_df)){
    df_food_insecurity  <-  survey_df |>
      dplyr::filter(question_concept_id %in% c(40192426, 40192517)) |> # 2 specific items
      # 40192426 = Within the past 12 months, were you worried whether the food you had bought just didn't last and
      #            you didn't have money to get more?
      # 40192517 = Within the past 12 months, were you worried whether your food would run out before you got money
      #            to buy more?
      dplyr::select(person_id, question_concept_id, answer_concept_id) |> # map answer_concept_id to value
      dplyr::filter(answer_concept_id != 903096) |> # remove skips
      dplyr::group_by(person_id) |> # group by person_id and calculate score
      dplyr::mutate(food_insecurity = 40192508 %in% answer_concept_id | 40192488 %in% answer_concept_id,
                    # label as positive for food insecurity if answered positively to either question
                    # 40192508 = Often true
                    # 40192488 = Sometimes true
                    nrows = length(answer_concept_id)) |> # how many questions did the participant answer?
      dplyr::filter(nrows == 2 | food_insecurity == TRUE) |>
                          # include only participants who answered all 2 questions OR there is a positive
      dplyr::select(person_id, food_insecurity) |> # only 2 columns in the final result
      dplyr::distinct(person_id, .keep_all = TRUE) |> # remove duplicate rows
      dplyr::right_join(survey_df |> # include participants without scores as NA
                          dplyr::group_by(person_id) |>
                          dplyr::summarize(),
                        by = 'person_id') |>
      dplyr::ungroup() # ungroup
  }
  df_food_insecurity
}
```

Housing Insecurity / Instability

Housing Insecurity (df_housing_insecurity)

```
# creates a binary categorical variable with value TRUE or FALSE
# TRUE denotes that the participant is at risk or currently experiencing housing insecurity
# (i.e., moved 2 or more times in the past year)

calc_housing_insecurity <- function(survey_df) {
  if (!is.null(survey_df)){
    df_housing_insecurity  <-  survey_df |>
      dplyr::filter(question_concept_id == 40192441) |> # 1 specific item
      # 40192441 = In the last 12 months, how many times have you or your family moved from one home to another?
      #            Number of moves in past 12 months:
      dplyr::select(person_id, question_concept_id, answer) |> # map answer_concept_id to value
      dplyr::filter(answer != "PMI: Skip") |> # remove skips
      dplyr::mutate(housing_insecurity = dplyr::case_when(
        answer %in% c("0","1") ~ FALSE, # 0 or 1 moves = false for household insecurity
        TRUE ~ TRUE)) |> # any response greater than 1 = true for household insecurity
      dplyr::select(person_id, housing_insecurity) |> # only 2 columns in the final result
      dplyr::right_join(survey_df |> # include participants without data as NA
                          dplyr::group_by(person_id) |>
                          dplyr::summarize(),
                        by = 'person_id')
  }
  df_housing_insecurity
}
```

Number of Moves (df_num_moves)

```
# creates a numeric variable representing the number of moves in the past year

calc_num_moves <- function(survey_df) {
  if (!is.null(survey_df)){
    df_num_moves  <-  survey_df |>
      dplyr::filter(question_concept_id == 40192441) |> # 1 specific item
      # 40192441 = In the last 12 months, how many times have you or your family moved from one home to another?
      #            Number of moves in past 12 months:
      dplyr::select(person_id, question_concept_id, answer) |> # map answer_concept_id to value
      dplyr::filter(answer != "PMI: Skip") |> # remove skips
      dplyr::mutate(num_moves = as.numeric(answer)) |>
      dplyr::select(person_id, num_moves) |> # only 2 columns in the final result
      dplyr::right_join(survey_df |> # include participants without scores as NA
                          dplyr::group_by(person_id) |>
                          dplyr::summarize(),
                        by = 'person_id')
  }
  df_num_moves
}
```

Housing Quality

df_housing_quality

```
# creates a binary categorical variable with value TRUE or FALSE
# TRUE denotes that the participant is endorsing a housing need, (i.e., selected at least one problem)

calc_housing_quality <- function(survey_df) {
  if (!is.null(survey_df)){
    df_housing_quality  <-  survey_df |>
      dplyr::filter(question_concept_id == 40192402) |> # 1 specific item
      # 40192402 = Think about the place you live. Do you have problems with any of the following? Select all that apply.
      dplyr::select(person_id, question_concept_id, answer_concept_id) |> # map answer_concept_id to value
      dplyr::mutate(value = dplyr::case_when(
        answer_concept_id == 40192392 ~ 0, # None of the above
        answer_concept_id == 903096 ~ 999, # PMI: Skip
        TRUE ~ 1)) |> # any response besides none indicates a problem and is coded to 1
      dplyr::filter(value != 999) |> # remove skips
      dplyr::group_by(person_id) |> # group by person_id and calculate score
      dplyr::mutate(housing_quality = 1 %in% value) |> # did the participant note any problems
      dplyr::select(person_id, housing_quality) |> # only 2 columns in the final result
      dplyr::distinct(person_id, .keep_all = TRUE) |> # remove duplicate rows
      dplyr::right_join(survey_df |> # include participants without scores as NA
                          dplyr::group_by(person_id) |>
                          dplyr::summarize(),
                        by = 'person_id') |>
      dplyr::ungroup() # ungroup
  }
  df_housing_quality
}
```

Perceived Stress

Sum Scoring (df__stress__sum)

```r
# creates a numeric score with range 0-40
# sum of individual item scores
# higher scores indicate higher levels of perceived stress

calc_stress_sum <- function(survey_df) {
  if (!is.null(survey_df)){
    df_stress_sum  <-  survey_df |>
      dplyr::filter(question_concept_id %in% c(40192381, 40192396, 40192419, 40192445, 40192449, 40192452, 40192462,
                                               40192491, 40192506, 40192525)) |> # 10 specific items
      # 40192381 = In the last month, how often have you felt that you were unable to control the important things
      #            in your life?
      # 40192396 = In the last month, how often have you been angered because of things that were outside of your control?
      # 40192419 = In the last month, how often have you felt confident about your ability to handle your personal problems?
      # 40192445 = In the last month, how often have you felt that you were on top of things?
      # 40192449 = In the last month, how often have you been able to control irritations in your life?
      # 40192452 = In the last month, how often have you been upset because of something that happened unexpectedly?
      # 40192462 = In the last month, how often have you felt difficulties were piling up so high that you could not
      #            overcome them?
      # 40192491 = In the last month, how often have you felt nervous and "stressed"?
      # 40192506 = In the last month, how often have you found that you could not cope with all the things that you had
      #            to do?
      # 40192525 = In the last month, how often have you felt that things were going your way?
      dplyr::select(person_id, question_concept_id, answer_concept_id) |> # map answer_concept_id to value
      dplyr::mutate(value = dplyr::case_when(
        answer_concept_id == 40192465 ~ 0, # Never
        answer_concept_id == 40192430 ~ 1, # Almost Never
        answer_concept_id == 40192429 ~ 2, # Sometimes
        answer_concept_id == 40192477 ~ 3, # Fairly Often
        answer_concept_id == 40192424 ~ 4, # Very Often
        TRUE ~ 999)) |>
      dplyr::filter(value != 999) |> # remove skips
      dplyr::mutate(value = dplyr::case_when(
        # reverse score for 5 questions
        question_concept_id %in% c(40192419, 40192445, 40192449, 40192525, 40192528) ~ 4-value, # 4 -> 0, 3 -> 1 etc
        TRUE ~ value)) |>
```

```r
      dplyr::group_by(person_id) |> # group by person_id and calculate sum score
      dplyr::mutate(stress_sum = sum(value,
                                     na.rm = TRUE),
                    nrows = length(value)) |> # how many questions did the participant answer?
      dplyr::filter(nrows == 10) |> # include only participants who answered all 10 questions
      dplyr::select(person_id, stress_sum) |> # only 2 columns in the final result
      dplyr::distinct(person_id, .keep_all = TRUE) |> # remove duplicate rows
      dplyr::right_join(survey_df |> # include participants without scores as NA
                          dplyr::group_by(person_id) |>
                          dplyr::summarize(),
                        by = 'person_id') |>
      dplyr::ungroup() # ungroup
  }
  df_stress_sum
}
```

Categorical Scoring (df_stress_category)

```r
# creates an ordinal categorical variable with values 'Low', 'Moderate', 'High'
# 'Low' denotes perceived stress score 0-13
# 'Moderate' denotes perceived stress score 14-26
# 'High' denotes perceived stress score 27-40

calc_stress_category <- function(survey_df) {
  if (!is.null(survey_df)){
    df_stress_category  <-  survey_df |>
      dplyr::filter(question_concept_id %in% c(40192381, 40192396, 40192419, 40192445, 40192449, 40192452, 40192462,
                                               40192491, 40192506, 40192525)) |> # 10 specific items
      # 40192381 = In the last month, how often have you felt that you were unable to control the important things
      #            in your life?
      # 40192396 = In the last month, how often have you been angered because of things that were outside of your control?
      # 40192419 = In the last month, how often have you felt confident about your ability to handle your personal problems?
      # 40192445 = In the last month, how often have you felt that you were on top of things?
      # 40192449 = In the last month, how often have you been able to control irritations in your life?
      # 40192452 = In the last month, how often have you been upset because of something that happened unexpectedly?
      # 40192462 = In the last month, how often have you felt difficulties were piling up so high that you could not
      #            overcome them?
      # 40192491 = In the last month, how often have you felt nervous and "stressed"?
      # 40192506 = In the last month, how often have you found that you could not cope with all the things that you had
      #            to do?
      # 40192525 = In the last month, how often have you felt that things were going your way?
      dplyr::select(person_id, question_concept_id, answer_concept_id) |> # map answer_concept_id to value
      dplyr::mutate(value = dplyr::case_when(
        answer_concept_id == 40192465 ~ 0, # Never
        answer_concept_id == 40192430 ~ 1, # Almost Never
        answer_concept_id == 40192429 ~ 2, # Sometimes
        answer_concept_id == 40192477 ~ 3, # Fairly Often
        answer_concept_id == 40192424 ~ 4, # Very Often
        TRUE ~ 999)) |>
      dplyr::filter(value != 999) |> # remove skips
      dplyr::mutate(value = dplyr::case_when(
        # reverse score for 5 questions
        question_concept_id %in% c(40192419, 40192445, 40192449, 40192525, 40192528) ~ 4-value, # 4 -> 0, 3 -> 1 etc
        TRUE ~ value)) |>
      dplyr::group_by(person_id) |> # group by person_id and calculate sum score
```

```r
      dplyr::mutate(stress_sum = sum(value,
                                       na.rm = TRUE),
                    nrows = length(value)) |> # how many questions did the participant answer?
      dplyr::filter(nrows == 10) |> # include only participants who answered all 10 questions
      dplyr::mutate(stress_category = dplyr::case_when(
        stress_sum < 14 ~ "Low", # 0-13 = low
        stress_sum < 27 ~ "Moderate", # 14-26 = moderate
        TRUE ~ "High")) |> # 27-40 = high
      dplyr::select(person_id, stress_category) |> # only 2 columns in the final result
      dplyr::distinct(person_id, .keep_all = TRUE) |> # remove duplicate rows
      dplyr::right_join(survey_df |> # include participants without scores as NA
                          dplyr::group_by(person_id) |>
                          dplyr::summarize(),
                        by = 'person_id') |>
      dplyr::ungroup() # ungroup
  }
  df_stress_category
}
```

Daily Spiritual Experiences

df_spirit

```
# creates a numeric score with range 6-36
# sum of individual item scores
# higher scores indicate more daily religious or spiritual experiences

calc_spirit <- function(survey_df) {
  if (!is.null(survey_df)){
    df_spirit  <-  survey_df |>
      dplyr::filter(question_concept_id %in% c(40192401, 40192415, 40192443, 40192471,
                                              40192475, 40192498)) |> # 6 specific items
      # 40192401 = How often do you feel deep inner peace or harmony?
      # 40192415 = How often do you feel that you are spiritually touched by the beauty of creation?
      # 40192443 = How often do you desire to be closer to or in union with God (or a higher power)?
      # 40192471 = How often do you feel God's (or a higher power's) love for you, directly or through others?
      # 40192475 = How often do you find strength and comfort in your religion?
      # 40192498 = How often do you feel God's (or a higher power's) presence?
      dplyr::select(person_id, question_concept_id, answer_concept_id) |> # map answer_concept_id to value
      dplyr::mutate(value = dplyr::case_when(
        answer_concept_id == 40192487 ~ 1, # I do not believe in God (or a higher power)
        answer_concept_id == 40192432 ~ 1, # I am not religious
        answer_concept_id == 40192509 ~ 1, # Never or almost never
        answer_concept_id == 40192459 ~ 2, # Once in a while
        answer_concept_id == 40192513 ~ 3, # Some days
        answer_concept_id == 40192484 ~ 4, # Most days
        answer_concept_id == 40192385 ~ 5, # Every day
        answer_concept_id == 40192403 ~ 6, # Many times a day
        TRUE ~ 999)) |>
      dplyr::filter(value != 999) |> # remove skips
      dplyr::group_by(person_id) |> # group by person_id and calculate sum score
      dplyr::mutate(spirit = sum(value,
                                 na.rm = TRUE),
                    nrows = length(value)) |> # how many questions did the participant answer?
      dplyr::filter(nrows == 6) |> # include only participants who answered all 6 questions
      dplyr::select(person_id, spirit) |> # only 2 columns in the final result
      dplyr::distinct(person_id, .keep_all = TRUE) |> # remove duplicate rows
      dplyr::right_join(survey_df |> # include participants without scores as NA
```

```
                              dplyr::group_by(person_id) |>
                              dplyr::summarize(),
                            by = 'person_id') |>
      dplyr::ungroup() # ungroup
  }
  df_spirit
}
```

Religious Service Attendance

df_religious_attendance

```r
# creates an ordinal categorical indicating the frequency of attending religious meetings or services

calc_religious_attendance <- function(survey_df) {
  if (!is.null(survey_df)){
    df_religious_attendance  <-  survey_df |>
      dplyr::filter(question_concept_id == 40192470) |> # 1 specific item
      # 40192470 = How often do you go to religious meetings or services?
      dplyr::select(person_id, question_concept_id, answer) |> # map answer_concept_id to value
      dplyr::filter(answer != "PMI: Skip") |> # remove skips
      dplyr::mutate(religious_attendance = answer) |>
      dplyr::select(person_id, religious_attendance) |> # only 2 columns in the final result
      dplyr::distinct(person_id, .keep_all = TRUE) |> # remove duplicate rows
      dplyr::right_join(survey_df |> # include participants without scores as NA
                          dplyr::group_by(person_id) |>
                          dplyr::summarize(),
                        by = 'person_id')
  }
  df_religious_attendance
}
```

English Proficiency

Speaks Other Language at Home (df_other_language)

```
# creates a nominal categorical variable with values 'Yes', 'No', or 'PMI: Prefer Not To Answer'
# 'Yes' denotes that the participant speaks a language other than English at home

calc_other_language <- function(survey_df) {
  if (!is.null(survey_df)){
    df_other_language  <-  survey_df |>
      dplyr::filter(question_concept_id == 40192526) |> # 1 specific item
      # 40192526 = Do you speak a language other than English at home?
      dplyr::select(person_id, question_concept_id, answer) |> # map answer_concept_id to value
      dplyr::filter(answer != "PMI: Skip") |> # remove skips
      dplyr::mutate(other_language = answer) |>
      dplyr::select(person_id, other_language) |> # only 2 columns in the final result
      dplyr::distinct(person_id, .keep_all = TRUE) |> # remove duplicate rows
      dplyr::right_join(survey_df |> # include participants without scores as NA
                          dplyr::group_by(person_id) |>
                          dplyr::summarize(),
                        by = 'person_id')
  }
  df_other_language
}
```

Level of English Proficiency (df_english_level)

```
# creates an ordinal categorical variable describing level of proficiency in English
# for participants who endorsed speaking a language other than English at home

calc_english_level <- function(survey_df) {
  if (!is.null(survey_df)){
    df_english_level  <-  survey_df |>
      dplyr::filter(question_concept_id == 40192529) |> # 1 specific item
      # 40192529 = Since you speak a language other than English at home, we are interested in your own thoughts about
      #            how well you think you speak English. Would you say you speak English...
      dplyr::select(person_id, question_concept_id, answer) |> # map answer_concept_id to value
      dplyr::filter(answer != "PMI: Skip") |> # remove skips
      dplyr::mutate(english_level = answer) |>
      dplyr::select(person_id, english_level) |> # only 2 columns in the final result
      dplyr::distinct(person_id, .keep_all = TRUE) |> # remove duplicate rows
      dplyr::right_join(survey_df |> # include participants without scores as NA
                          dplyr::group_by(person_id) |>
                          dplyr::summarize(),
                        by = 'person_id')
  }
  df_english_level
}
```

English Proficient (df_english_proficient)

```
# creates a nominal categorical variable with values 'Proficient', 'Not proficient', or 'Unknown' for participants who
# endorsed speaking a language other than English at home
# 'Proficient' denotes participants who endorsed speaking English 'Very well' or 'Well'
# 'Not proficient' denotes participants who endorsed speaking English 'Not well' or 'Not at all'

calc_english_proficient <- function(survey_df) {
  if (!is.null(survey_df)){
    df_english_proficient  <-  survey_df |>
      dplyr::filter(question_concept_id == 40192529) |> # 1 specific item
      # 40192529 = Since you speak a language other than English at home, we are interested in your own thoughts about
      #            how well you think you speak English. Would you say you speak English...
      dplyr::select(person_id, question_concept_id, answer_concept_id) |> # map answer_concept_id to value
      dplyr::mutate(english_proficient = dplyr::case_when(
        answer_concept_id == 40192435 ~ "Proficient", # Very well
        answer_concept_id == 40192510 ~ "Proficient", # Well
        answer_concept_id == 40192405 ~ "Not proficient", # Not well
        answer_concept_id == 40192387 ~ "Not proficient", # Not at all
        answer_concept_id == 903087 ~ "Unknown", # PMI: Dont Know
        answer_concept_id == 903079 ~ "Unknown", # PMI: Prefer Not To Answer
        TRUE ~ "none")) |>
      dplyr::filter(english_proficient != "none") |> # remove skips
      dplyr::select(person_id, english_proficient) |> # only 2 columns in the final result
      dplyr::distinct(person_id, .keep_all = TRUE) |> # remove duplicate rows
      dplyr::right_join(survey_df |> # include participants without scores as NA
                          dplyr::group_by(person_id) |>
                          dplyr::summarize(),
                        by = 'person_id') |>
      dplyr::ungroup() # ungroup
  }
  df_english_proficient
}
```
