# Supplementary material for: User guide for Social Determinants of Health Survey data in the All of Us Research Program
Source: J Am Med Inform Assoc. 2024 Aug 27;31(12):3032–41. doi: 10.1093/jamia/ocae214 (PMC11631056; doi:10.1093/jamia/ocae214)
Supplement: ocae214_Supplementary_Data [file ocae214_supplementary_data.zip › ocae214_Supplementary_Data/2024_06_26_Supplemental_File_6_Python_Functions_PDF.pdf]

All of Us User Guide - Python Functions

Neighborhood Cohesion

df_cohesion

```
# creates a numeric score with range 1-5
# mean of individual item scores
# higher scores indicate higher neighborhood cohesion

import pandas as pd
import numpy as np

def calc_cohesion(survey_df):
    if survey_df is not None and not survey_df.empty:
        filter_questions = [40192463, 40192411, 40192499, 40192417] # 4 specific items
        df_cohesion = survey_df[survey_df['question_concept_id'].isin(filter_questions)]
        # 40192463 = How much you agree or disagree that people around here are willing to help their neighbor?
        # 40192411 = How much you agree or disagree that people in your neighborhood generally get along with each other?
        # 40192499 = How much you agree or disagree that people in your neighborhood can be trusted?
        # 40192417 = How much you agree or disagree that people in your neighborhood share the same values?

        # map answer_concept_id to value
        score_mapping = {
            40192514: 5,  # Strongly agree
            40192455: 4,  # Agree
            40192524: 3,  # Neutral (neither agree nor disagree)
            40192408: 2,  # Disagree
            40192422: 1,  # Strongly disagree
        }
        df_cohesion['value'] = df_cohesion['answer_concept_id'].map(score_mapping).fillna(999)
        df_filtered = df_cohesion[df_cohesion['value'] != 999] # remove skips
```

```python
        # group by person_id and calculate mean score
        counts_per_person = df_filtered.groupby('person_id')['question_concept_id'].nunique().reset_index(name=
                                                                                                         'answered_questions')
        df_filtered = pd.merge(df_filtered, counts_per_person, on='person_id')

        df_filtered = df_filtered[df_filtered['answered_questions'] == len(filter_questions)]
                                              # include only participants who answered all 4 questions
        df_cohesion = df_filtered.groupby('person_id')['value'].mean().reset_index()

        df_cohesion.columns = ['person_id', 'cohesion']
        df_cohesion['cohesion'] = df_cohesion['cohesion'].round(2)  # round to 2 decimals

        # include participants without scores as NA
        df_final = pd.merge(survey_df[['person_id']].drop_duplicates(), df_cohesion, on='person_id', how='left')

        return df_final
    else:
        return None

df_cohesion = calc_cohesion(survey_df)
```

Neighborhood Disorder

Neighborhood Disorder (df_disorder)

```
# creates a numeric score with range 1-4
# mean of individual item scores
# higher scores indicate higher neighborhood disorder, while lower scores indicate order

import pandas as pd
import numpy as np

def calc_disorder(survey_df):
    if survey_df is not None and not survey_df.empty:
        question_ids = [40192420, 40192522, 40192412, 40192469, 40192456, 40192386,
                        40192500, 40192493, 40192457, 40192476, 40192404, 40192400, 40192384] # 13 specific items
        df_filtered = survey_df[survey_df['question_concept_id'].isin(question_ids)]
        # 40192420 = How much you agree or disagree that there is a lot of graffiti in your neighborhood?
        # 40192522 = How much you agree or disagree that your neighborhood is noisy?
        # 40192412 = How much you agree or disagree that vandalism is common in your neighborhood?
        # 40192469 = How much you agree or disagree that there are lot of abandoned buildings in your neighborhood?
        # 40192456 = How much you agree or disagree that your neighborhood is clean?
        # 40192386 = How much you agree or disagree that people in your neighborhood take good care of their houses
        #            and apartments?
        # 40192500 = How much you agree or disagree that there are too many people hanging around on the streets near
        #            your home?
        # 40192493 = How much you agree or disagree that there is a lot of crime in your neighborhood?
        # 40192457 = How much you agree or disagree that there is too much drug use in your neighborhood?
        # 40192476 = How much you agree or disagree that there is too much alcohol use in your neighborhood?
        # 40192404 = How much you agree or disagree that you are always having trouble with your neighbors?
        # 40192400 = How much you agree or disagree that in your neighborhood people watch out for each other?
        # 40192384 = How much you agree or disagree that your neighborhood is safe?

        # map answer_concept_id to value
        score_mapping = {
            40192514: 4,  # Strongly agree
            40192455: 3,  # Agree
            40192408: 2,  # Disagree
            40192422: 1,  # Strongly disagree
        }
```

```
        df_filtered['value'] = df_filtered['answer_concept_id'].map(score_mapping).fillna(999)
        df_filtered = df_filtered[df_filtered['value'] != 999]  # remove skips

        reverse_questions = [40192456, 40192386, 40192400, 40192384] # Reverse scoring for certain questions
        df_filtered['value'] = df_filtered.apply(
          lambda row: 5 - row['value'] if row['question_concept_id'] in reverse_questions else row['value'], axis=1)

        # group by person_id and calculate mean score
        counts_per_person = df_filtered.groupby('person_id')['question_concept_id'].nunique().reset_index(name=
                                                                                    'answered_questions')
        df_filtered = pd.merge(df_filtered, counts_per_person, on='person_id')

        df_filtered = df_filtered[df_filtered['answered_questions'] == len(question_ids)]
                                            # include only participants who answered all 13 questions
        df_disorder = df_filtered.groupby('person_id')['value'].mean().reset_index()

        df_disorder.columns = ['person_id', 'disorder']
        df_disorder['disorder'] = df_disorder['disorder'].round(2)  # round to 2 decimals

        # include participants without scores as NA
        df_final = pd.merge(survey_df[['person_id']].drop_duplicates(), df_disorder, on='person_id', how='left')

        return df_final
    else:
        return None

df_disorder = calc_disorder(survey_df)
```

Neighborhood Physical Disorder (df_physical_disorder)

```
# creates a numeric score with range 1-4
# mean of individual item scores
# higher scores indicate higher neighborhood physical disorder, while lower scores indicate physical order

import pandas as pd
import numpy as np

def calc_physical_disorder(survey_df):
    if survey_df is not None and not survey_df.empty:
        question_ids = [40192420, 40192522, 40192412, 40192469, 40192456, 40192386] # 6 specific items
        df_filtered = survey_df[survey_df['question_concept_id'].isin(question_ids)]
        # 40192420 = How much you agree or disagree that there is a lot of graffiti in your neighborhood?
        # 40192522 = How much you agree or disagree that your neighborhood is noisy?
        # 40192412 = How much you agree or disagree that vandalism is common in your neighborhood?
        # 40192469 = How much you agree or disagree that there are lot of abandoned buildings in your neighborhood?
        # 40192456 = How much you agree or disagree that your neighborhood is clean?
        # 40192386 = How much you agree or disagree that people in your neighborhood take good care of their houses
        #            and apartments?

        # map answer_concept_id to value
        score_mapping = {
            40192514: 4,  # Strongly agree
            40192455: 3,  # Agree
            40192408: 2,  # Disagree
            40192422: 1,  # Strongly disagree
        }
        df_filtered['value'] = df_filtered['answer_concept_id'].map(score_mapping).fillna(999)
        df_filtered = df_filtered[df_filtered['value'] != 999]  # remove skips

        reverse_questions = [40192456, 40192386] # Reverse scoring for certain questions
        df_filtered['value'] = df_filtered.apply(
          lambda row: 5 - row['value'] if row['question_concept_id'] in reverse_questions else row['value'], axis=1)

        # group by person_id and calculate mean score
        counts_per_person = df_filtered.groupby('person_id')['question_concept_id'].nunique().reset_index(name=
                                                                                                 'answered_questions')
        df_filtered = pd.merge(df_filtered, counts_per_person, on='person_id')
```

```
        df_filtered = df_filtered[df_filtered['answered_questions'] == len(question_ids)]
                                              # include only participants who answered all 6 questions
        df_physical_disorder = df_filtered.groupby('person_id')['value'].mean().reset_index()

        df_physical_disorder.columns = ['person_id', 'physical_disorder']
        df_physical_disorder['physical_disorder'] = df_physical_disorder['physical_disorder'].round(2)  # round to 2 decimals

        # include participants without scores as NA
        df_final = pd.merge(survey_df[['person_id']].drop_duplicates(), df_physical_disorder, on='person_id', how='left')

        return df_final
    else:
        return None

df_physical_disorder = calc_physical_disorder(survey_df)
```

Neighborhood Social Disorder (df_social_disorder)

```
# creates a numeric score with range 1-4
# mean of individual item scores
# higher scores indicate higher neighborhood social disorder, while lower scores indicate social order

import pandas as pd
import numpy as np

def calc_social_disorder(survey_df):
    if survey_df is not None and not survey_df.empty:
        question_ids = [40192500, 40192493, 40192457, 40192476, 40192404, 40192400, 40192384] # 7 specific items
        df_filtered = survey_df[survey_df['question_concept_id'].isin(question_ids)]
        # 40192500 = How much you agree or disagree that there are too many people hanging around on the streets near
        #            your home?
        # 40192493 = How much you agree or disagree that there is a lot of crime in your neighborhood?
        # 40192457 = How much you agree or disagree that there is too much drug use in your neighborhood?
        # 40192476 = How much you agree or disagree that there is too much alcohol use in your neighborhood?
        # 40192404 = How much you agree or disagree that you are always having trouble with your neighbors?
        # 40192400 = How much you agree or disagree that in your neighborhood people watch out for each other?
        # 40192384 = How much you agree or disagree that your neighborhood is safe?

        # map answer_concept_id to value
        score_mapping = {
            40192514: 4,  # Strongly agree
            40192455: 3,  # Agree
            40192408: 2,  # Disagree
            40192422: 1,  # Strongly disagree
        }
        df_filtered['value'] = df_filtered['answer_concept_id'].map(score_mapping).fillna(999)
        df_filtered = df_filtered[df_filtered['value'] != 999]  # remove skips

        reverse_questions = [40192400, 40192384] # Reverse scoring for certain questions
        df_filtered['value'] = df_filtered.apply(
          lambda row: 5 - row['value'] if row['question_concept_id'] in reverse_questions else row['value'], axis=1)

        # group by person_id and calculate mean score
        counts_per_person = df_filtered.groupby('person_id')['question_concept_id'].nunique().reset_index(name=
                                                                                            'answered_questions')
```

```
        df_filtered = pd.merge(df_filtered, counts_per_person, on='person_id')

        df_filtered = df_filtered[df_filtered['answered_questions'] == len(question_ids)]
                                              # include only participants who answered all 7 questions
        df_social_disorder = df_filtered.groupby('person_id')['value'].mean().reset_index()

        df_social_disorder.columns = ['person_id', 'social_disorder']
        df_social_disorder['social_disorder'] = df_social_disorder['social_disorder'].round(2)  # round to 2 decimals

        # include participants without scores as NA
        df_final = pd.merge(survey_df[['person_id']].drop_duplicates(), df_social_disorder, on='person_id', how='left')

        return df_final
    else:
        return None

df_social_disorder = calc_social_disorder(survey_df)
```

Neighborhood Environment

Residential Density (df_density)

```
# creates a binary categorical variable with value 'High' or 'Low'
# 'Low' denotes low residential density (i.e., detached single family housing)
# 'High' denotes high residential density

import pandas as pd

def calc_density(survey_df):
    if survey_df is not None and not survey_df.empty:
        df_filtered = survey_df[survey_df['question_concept_id'] == 40192458] # 1 specific item
        # 40192458=What is the main type of housing in your neighborhood?

        # map answer_concept_id to value
        scoring_map = {
            40192407: "Low",  # Detached single-family housing indicates low residential density
            903087: "none",   # Don't Know - considered non-answers
            903096: "none",   # Skip - considered non-answers
            40192520: "none"  # Does not apply - considered non-answers
        }
        df_filtered['density'] = df_filtered['answer_concept_id'].map(scoring_map).fillna("High")
        df_filtered = df_filtered[df_filtered['density'] != "none"] # remove skips

        df_filtered = df_filtered[['person_id', 'density']].drop_duplicates()

        # include participants without scores as NA
        df_density = pd.merge(survey_df[['person_id']].drop_duplicates(), df_filtered, on='person_id', how='left')

        return df_density

df_density = calc_density(survey_df)
```

Environmental Support for Physical Activity (df_spa)

```
# creates a numeric score with range 7-28
# sum of individual item scores
# higher scores indicate greater environmental support for physical activity

import pandas as pd

def calc_spa(survey_df):
    if survey_df is not None and not survey_df.empty:
        question_ids = [40192436, 40192440, 40192437, 40192431, 40192410, 40192492, 40192414] # 7 specific items
        df_filtered = survey_df[survey_df['question_concept_id'].isin(question_ids)]
        # 40192436 = Many shops, stores, markets or other places to buy things I need are within easy walking distance
        #            of my home. Would you say that you...
        # 40192440 = It is within a 10-15 minute walk to a transit stop (such as bus, train, trolley, or tram) from my home.
        #            Would you say that you...
        # 40192437 = There are sidewalks on most of the streets in my neighborhood. Would you say that you...
        # 40192431 = There are facilities to bicycle in or near my neighborhood, such as special lanes, separate paths or
        #            trails, or shared use paths for cycles and pedestrians. Would you say that you...
        # 40192410 = My neighborhood has several free or low-cost recreation facilities, such as parks, walking trails,
        #            bike paths, recreation centers, playgrounds, public swimming pools, etc. Would you say that you...
        # 40192492 = The crime rate in my neighborhood makes it unsafe to go on walks at night. Would you say that you...
        # 40192414 = The crime rate in my neighborhood makes it unsafe to go on walks during the day. Would you say that
        #            you...

        # map answer_concept_id to value
        score_mapping = {
            40192514: 4, # Strongly agree
            40192478: 3, # Somewhat agree
            40192527: 2, # Somewhat disagree
            40192422: 1, # Strongly disagree
        }
        df_filtered['value'] = df_filtered['answer_concept_id'].map(score_mapping).fillna(999)
        df_filtered = df_filtered[df_filtered['value'] != 999] # remove skips

        reverse_score_questions = [40192492, 40192414]  # Reverse score for specific questions
        df_filtered['value'] = df_filtered.apply(
            lambda row: 5 - row['value'] if row['question_concept_id'] in reverse_score_questions else row['value'],
            axis=1)
```

```python
        # group by person_id and calculate sum score
        counts_per_person = df_filtered.groupby('person_id')['question_concept_id'].nunique().reset_index(name=
                                                                                                      'answered_questions')
        df_filtered = pd.merge(df_filtered, counts_per_person, on='person_id')

        df_filtered = df_filtered[df_filtered['answered_questions'] == len(question_ids)]
                                              # include only participants who answered all 7 questions
        spa_scores = df_filtered.groupby('person_id')['value'].sum().reset_index()

        spa_scores.columns = ['person_id', 'spa']

        # include participants without scores as NA
        df_spa = pd.merge(survey_df[['person_id']].drop_duplicates(), spa_scores, on='person_id', how='left')
        df_spa['spa'] = df_spa['spa'].round(2)  # round to 2 decimals

        return df_spa

df_spa = calc_spa(survey_df)
```

Crime Safety (df_crime_safety)

```
# creates a numeric score with range 1-4
# mean of individual item scores
# higher scores indicate higher crime safety

import pandas as pd

def calc_crime_safety(survey_df):
    if survey_df is not None:
        question_ids = [40192414, 40192492] # 2 specific items
        df_filtered = survey_df[survey_df['question_concept_id'].isin(question_ids)]
        # 40192414 = The crime rate in my neighborhood makes it unsafe to go on walks during the day. Would you say that you...
        # 40192492 = The crime rate in my neighborhood makes it unsafe to go on walks at night. Would you say that you...

        # map answer_concept_id to value, reverse code for 2 questions
        score_mapping = {40192514: 1,  # Strongly agree
                         40192478: 2,  # Somewhat agree
                         40192527: 3,  # Somewhat disagree
                         40192422: 4,  # Strongly disagree
        }
        df_filtered['value'] = df_filtered['answer_concept_id'].map(score_mapping).fillna(999)
        df_filtered = df_filtered[df_filtered['value'] != 999] # remove skips

        # group by person_id and calculate mean score
        df_filtered['count'] = df_filtered.groupby('person_id')['value'].transform('count')
        df_filtered = df_filtered[df_filtered['count'] == len(question_ids)]
                                              # include only participants who answered all 2 questions
        df_crime_safety = df_filtered.groupby('person_id')['value'].mean().reset_index(name='crime_safety')

        # include participants without scores as NA
        df_final = pd.merge(survey_df[['person_id']].drop_duplicates(), df_crime_safety, on='person_id', how='left')
        df_crime_safety['crime_safety'] = df_crime_safety['crime_safety'].round(2)  # round to 2 decimals

        return df_final
    else:
        return pd.DataFrame()

df_crime_safety = calc_crime_safety(survey_df)
```

Neighborhood Environment Index (df_nei)

```python
# creates a numeric score with range 0-6
# sum of individual item scores
# higher scores indicate a more favorable built environment for physical activity

import pandas as pd

def calc_nei(survey_df):
    if survey_df is not None and not survey_df.empty:
        question_ids = [40192410, 40192431, 40192436, 40192437, 40192440, 40192458] # 6 specific items
        df_filtered = survey_df[survey_df['question_concept_id'].isin(question_ids)]
        # 40192410 = My neighborhood has several free or low-cost recreation facilities, such as parks, walking trails,
        #            bike paths, recreation centers, playgrounds, public swimming pools, etc. Would you say that you...
        # 40192431 = There are facilities to bicycle in or near my neighborhood, such as special lanes, separate paths or
        #            trails, or shared use paths for cycles and pedestrians. Would you say that you...
        # 40192436 = Many shops, stores, markets or other places to buy things I need are within easy walking distance of
        #            my home. Would you say that you...
        # 40192437 = There are sidewalks on most of the streets in my neighborhood. Would you say that you...
        # 40192440 = It is within a 10-15 minute walk to a transit stop (such as bus, train, trolley, or tram) from my home.
        #            Would you say that you...
        # 40192458 = What is the main type of housing in your neighborhood?

        # map answer_concept_id to value
        score_mapping = {
            40192527: 0, # Somewhat disagree
            40192422: 0, # Strongly disagree
            40192407: 0, # Detached single-family housing indicates low density (scored as 0 here)
        }
        df_filtered['value'] = df_filtered['answer_concept_id'].map(score_mapping).fillna(1)
        non_response_values = [903087, 903096, 40192520]  # PMI: Don't Know, PMI: Skip, Does not apply
        df_filtered = df_filtered[~df_filtered['answer_concept_id'].isin(non_response_values)] # remove skips

        # group by person_id and calculate sum score
        df_nei = df_filtered.groupby('person_id')['value'].sum().reset_index(name='nei')
        counts_per_person = df_filtered.groupby('person_id')['question_concept_id'].nunique().reset_index(name=
                                                                                                   'answered_questions')
        df_nei = pd.merge(df_nei, counts_per_person, on='person_id')
```

```
        df_nei = df_nei[df_nei['answered_questions'] == len(question_ids)]
                                # include only participants who answered all 6 questions

        # include participants without scores as NA
        df_final = pd.merge(survey_df[['person_id']].drop_duplicates(), df_nei[['person_id', 'nei']], on='person_id',
                                                                                                  how='left')
        df_nei['nei'] = df_nei['nei'].round(2)  # round to 2 decimals

        return df_final
    else:
        return pd.DataFrame()

df_nei = calc_nei(survey_df)
```

Social Support

Overall Social Support (df_social_support)

```
# creates a numeric score with range 0-100
# mean of individual item scores transformed to a 0-100 scale
# higher scores indicate more social support

import pandas as pd

def calc_social_support(survey_df):
    if survey_df is not None:
        question_ids = [40192388, 40192399, 40192439, 40192442, 40192446, 40192480, 40192511, 40192528] # 8 specific items
        df_filtered = survey_df[survey_df['question_concept_id'].isin(question_ids)]
        # 40192388 = How often do you have someone to prepare your meals if you were unable to do it yourself?
        # 40192399 = How often do you have someone who understands your problems?
        # 40192439 = How often do you have someone to have a good time with?
        # 40192442 = How often do you have someone to help you if you were confined to bed?
        # 40192446 = How often do you have someone to love and make you feel wanted?
        # 40192480 = How often do you have someone to take you to the doctor if you need it?
        # 40192511 = How often do you have someone to help you with daily chores if you were sick?
        # 40192528 = How often do you have someone to turn to for suggestions about how to deal with a personal problem?

        # map answer_concept_id to value
        score_mapping = {
            40192454: 1,  # None of the time
            40192518: 2,  # A little of the time
            40192486: 3,  # Some of the time
            40192382: 4,  # Most of the time
            40192521: 5,  # All of the time
        }
        df_filtered['value'] = df_filtered['answer_concept_id'].map(score_mapping).fillna(999)
        df_filtered = df_filtered[df_filtered['value'] != 999] # remove skips

        # group by person_id and calculate mean score
        df_filtered['count'] = df_filtered.groupby('person_id')['value'].transform('count')
        df_filtered = df_filtered[df_filtered['count'] == len(question_ids)]
                                              # include only participants who answered all 8 questions
```

```
        df_social_support = df_filtered.groupby('person_id')['value'].apply(
            lambda x: round(100 * (x.sum() - 8) / (40 - 8), 2)
        ).reset_index(name='social_support')  # Calculate social support score

        # include participants without scores as NA
        df_final = pd.merge(survey_df[['person_id']].drop_duplicates(), df_social_support[['person_id', 'social_support']],
                                                                        on='person_id', how='left')
        df_social_support['social_support'] = df_social_support['social_support'].round(2)  # round to 2 decimals

        return df_final
    else:
        return pd.DataFrame()

df_social_support = calc_social_support(survey_df)
```

Instrumental Subscale (df_ins_support)

```
# creates a numeric score with range 0-100
# mean of individual item scores transformed to a 0-100 scale
# higher scores indicate more tangible support

import pandas as pd

def calc_ins_support(survey_df):
    if survey_df is not None:
        question_ids = [40192388, 40192442, 40192480, 40192511] # 4 specific items
        df_filtered = survey_df[survey_df['question_concept_id'].isin(question_ids)]
        # 40192388 = How often do you have someone to prepare your meals if you were unable to do it yourself?
        # 40192442 = How often do you have someone to help you if you were confined to bed?
        # 40192480 = How often do you have someone to take you to the doctor if you need it?
        # 40192511 = How often do you have someone to help you with daily chores if you were sick?

        # map answer_concept_id to value
        score_mapping = {
            40192454: 1,  # None of the time
            40192518: 2,  # A little of the time
            40192486: 3,  # Some of the time
            40192382: 4,  # Most of the time
            40192521: 5,  # All of the time
        }
        df_filtered['value'] = df_filtered['answer_concept_id'].map(score_mapping).fillna(999)
        df_filtered = df_filtered[df_filtered['value'] != 999] # remove skips

        # group by person_id and calculate mean score
        df_filtered['count'] = df_filtered.groupby('person_id')['value'].transform('count')
        df_filtered = df_filtered[df_filtered['count'] == len(question_ids)]
                                              # include only participants who answered all 4 questions

        df_filtered['ins_support'] = df_filtered.groupby('person_id')['value'].transform(
          lambda x: (100 * (x.sum() - x.count() * 1) / ((x.count() * 5) - x.count() * 1))
          if x.count() == len(question_ids) else None)

        df_ins_support = df_filtered[['person_id', 'ins_support']].drop_duplicates(subset=['person_id'])
```

```
        # include participants without scores as NA
        df_final = pd.merge(survey_df[['person_id']].drop_duplicates(), df_ins_support, on='person_id', how='left')
        df_ins_support['ins_support'] = df_ins_support['ins_support'].round(2)  # round to 2 decimals

        return df_final
    else:
        return pd.DataFrame()

df_ins_support = calc_ins_support(survey_df)
```

Emotional Subscale (df_emo_support)

```python
# creates a numeric score with range 0-100
# mean of individual item scores transformed to a 0-100 scale
# higher scores indicate more emotional support

import pandas as pd

def calc_emo_support(survey_df):
    if survey_df is not None:
        question_ids = [40192399, 40192439, 40192446, 40192528] # 4 specific items
        df_filtered = survey_df[survey_df['question_concept_id'].isin(question_ids)]
        # 40192399 = How often do you have someone who understands your problems?
        # 40192439 = How often do you have someone to have a good time with?
        # 40192446 = How often do you have someone to love and make you feel wanted?
        # 40192528 = How often do you have someone to turn to for suggestions about how to deal with a personal problem?

        # map answer_concept_id to value
        score_mapping = {
            40192454: 1,  # None of the time
            40192518: 2,  # A little of the time
            40192486: 3,  # Some of the time
            40192382: 4,  # Most of the time
            40192521: 5,  # All of the time
        }

        df_filtered['values'] = df_filtered['answer_concept_id'].map(score_mapping).fillna(999)
        df_filtered = df_filtered[df_filtered['values'] != 999] # remove skips

        # group by person_id and calculate score
        df_grouped = df_filtered.groupby('person_id')['values'].agg(sum='sum', count='count')
        df_grouped = df_grouped[df_grouped['count'] == len(question_ids)]
                                           # include only participants who answered all 4 questions

        # Calculate the emotional support score on a 0-100 scale
        df_grouped['emo_support'] = round(
          (df_grouped['sum'] - df_grouped['count']) * 100 / ((5 - 1) * df_grouped['count'])
          , 2)
```

```python
        # include participants without scores as NA
        df_final = pd.merge(survey_df[['person_id']].drop_duplicates(), df_grouped[['emo_support']],
                                                left_on='person_id', right_index=True, how='left')
        df_final['emo_support'] = df_final['emo_support'].round(2) # round to 2 decimals

        return df_final

df_emo_support = calc_emo_support(survey_df)
```

Loneliness

df_loneliness

```
# creates a numeric score with range 8-32
# sum of individual item scores
# higher scores indicate higher degree of loneliness

import pandas as pd

def calc_loneliness(survey_df):
    if survey_df is not None:
        question_ids = [40192390, 40192397, 40192398, 40192494, 40192501,
                        40192504, 40192507, 40192516] # 8 specific questions
        # 40192390 = How often do you feel that you are unhappy being so withdrawn?
        # 40192397 = How often do you feel that there is no one you can turn to?
        # 40192398 = How often do you feel left out?
        # 40192494 = How often do you feel that people are around you but not with you?
        # 40192501 = How often do you feel isolated from others?
        # 40192504 = How often do you feel that you are an outgoing person?
        # 40192507 = How often do you feel lack companionship?
        # 40192516 = How often do you fell that you can find companionship when you want it?

        # map answer_concept_id to value
        score_mapping = {
            40192465: 1,  # Never
            40192481: 2,  # Rarely
            40192429: 3,  # Sometimes
            40192482: 4,  # Often
        }
        reverse_questions = [40192504, 40192516]

        df_filtered = survey_df[survey_df['question_concept_id'].isin(question_ids)]
        df_filtered['values'] = df_filtered['answer_concept_id'].map(score_mapping).fillna(999)

        df_filtered.loc[df_filtered['question_concept_id'].isin(reverse_questions), 'values'] = df_filtered['values'].apply(
          lambda x: 5-x if x != 999 else x)
        df_filtered = df_filtered[df_filtered['values'] != 999] # remove skips
```

```
        # group by person_id and calculate sum score
        df_grouped = df_filtered.groupby('person_id')['values'].agg(['sum', 'count'])
        df_grouped = df_grouped[df_grouped['count'] == len(question_ids)]
                                  # include only participants who answered all 8 questions

        df_loneliness = df_grouped.reset_index()
        df_loneliness['loneliness'] = df_loneliness['sum']

        # include participants without scores as NA
        df_final = pd.merge(survey_df[['person_id']].drop_duplicates(), df_loneliness[['person_id', 'loneliness']],
                                                                       on='person_id', how='left')
        df_loneliness['loneliness'] = df_loneliness['loneliness'].round(2)  # round to 2 decimals

        return df_final
    else:
        return pd.DataFrame()

df_loneliness = calc_loneliness(survey_df)
```

Perceived Everyday Discrimination

Situation-based Scoring (df_edd_situation)

```
# creates a numeric score with range 0-9
# indicates how many questions the participant responded to with something other than 'Never'
# higher scores indicate more frequent perceived experience of unfair treatment

# reason is an optional argument
# can limit to participants who provided a particular reason for discrimination, e.g. race or age
# see survey for options

import pandas as pd

def calc_edd_situation(survey_df, reason=None):
    if survey_df is not None and not survey_df.empty:
        question_ids = [40192380, 40192395, 40192416, 40192451, 40192466,
                        40192489, 40192490, 40192496, 40192519] # 9 specific items
        df_filtered = survey_df[survey_df['question_concept_id'].isin(question_ids)]
        # 40192380 = In your day-to-day life, how often do people act as if they are afraid of you?
        # 40192395 = In your day-to-day life, how often do people act as if they think you are dishonest?
        # 40192416 = In your day-to-day life, how often do you receive poorer service than other people at restaurants
        #            or stores?
        # 40192451 = In your day-to-day life, how often are you threatened or harassed?
        # 40192466 = In your day-to-day life, how often are you treated with less courtesy than other people?
        # 40192489 = In your day-to-day life, how often are you treated with less respect than other people?
        # 40192490 = In your day-to-day life, how often do people act as if they think you are not smart?
        # 40192496 = In your day-to-day life, how often do people act as if they're better than you are?
        # 40192519 = In your day-to-day life, how often are you called names or insulted?

        # map answer_concept_id to value
        score_mapping = {
            40192465: 0,  # Never
            903096: 999,  # PMI: Skip
        }
        df_filtered['value'] = df_filtered['answer_concept_id'].map(score_mapping).fillna(1)
                                                    # Default to 1 for all other responses
        df_filtered = df_filtered[df_filtered['value'] != 999] # remove skips
```

```
        # group by person_id and calculate sum score
        df_grouped = df_filtered.groupby('person_id')['value'].agg(['sum', 'count']).reset_index()
        df_grouped = df_grouped.rename(columns={'sum': 'edd_situation', 'count': 'nrows'})
        df_grouped = df_grouped[df_grouped['nrows'] == len(question_ids)]
                                   # include only participants who answered all 9 questions

        # include participants without scores as NA
        df_final = pd.merge(survey_df[['person_id']].drop_duplicates(), df_grouped[['person_id', 'edd_situation']],
                                                                        on='person_id', how='left')
        df_final['edd_situation'] = df_final['edd_situation'].round(2) # round to 2 decimals

        return df_final
    else:
        return pd.DataFrame()

df_edd_situation = calc_edd_situation(survey_df)
```

Frequency-based Scoring (df_edd_frequency)

```python
# creates a numeric score with range 9-54
# sum of individual item scores
# higher scores indicate more frequent perceived experience of unfair treatment

# reason is an optional argument
# can limit to participants who provided a particular reason for discrimination, e.g. race or age
# see survey for options

import pandas as pd

def calc_edd_frequency(survey_df, question_ids=None, score_mapping=None):
    if question_ids is None:
        question_ids = [40192380, 40192395, 40192416, 40192451, 40192466,
                        40192489, 40192490, 40192496, 40192519] # 9 specific items
        # 40192380 = In your day-to-day life, how often do people act as if they are afraid of you?
        # 40192395 = In your day-to-day life, how often do people act as if they think you are dishonest?
        # 40192416 = In your day-to-day life, how often do you receive poorer service than other people at restaurants
        #            or stores?
        # 40192451 = In your day-to-day life, how often are you threatened or harassed?
        # 40192466 = In your day-to-day life, how often are you treated with less courtesy than other people?
        # 40192489 = In your day-to-day life, how often are you treated with less respect than other people?
        # 40192490 = In your day-to-day life, how often do people act as if they think you are not smart?
        # 40192496 = In your day-to-day life, how often do people act as if they're better than you are?
        # 40192519 = In your day-to-day life, how often are you called names or insulted?

    # map answer_concept_id to value
    if score_mapping is None:
        score_mapping = {
            40192465: 1,  # Never
            40192464: 2,  # Less than once a year
            40192453: 3,  # A few times a year
            40192461: 4,  # A few times a month
            40192391: 5,  # At least once a week
            40192421: 6,  # Almost everyday
            903096: None  # PMI: Skip, exclude from scoring
        }
    df_filtered = survey_df[survey_df['question_concept_id'].isin(question_ids)]
```

```python
    df_filtered['frequency_score'] = df_filtered['answer_concept_id'].map(lambda x: score_mapping.get(x, None))
    df_filtered = df_filtered.dropna(subset=['frequency_score'])  # Exclude responses without a valid score

    # group by person_id and calculate sum score
    df_edd_frequency = df_filtered.groupby('person_id')['frequency_score'].agg(['sum', 'count']).reset_index()
    df_edd_frequency.columns = ['person_id', 'edd_frequency', 'answered_questions']
    df_edd_frequency = df_edd_frequency[df_edd_frequency['answered_questions'] == len(question_ids)]
                                                        # include only participants who answered all 9 questions

    # include participants without scores as NA
    df_final = pd.merge(survey_df[['person_id']].drop_duplicates(), df_edd_frequency[['person_id', 'edd_frequency']],
                                                                  on='person_id', how='left')
    df_final['edd_frequency'] = df_final['edd_frequency'].round(2)  # round to 2 decimals

    return df_final

df_edd_frequency = calc_edd_frequency(survey_df)
```

Chronicity-based Scoring (df_edd_chronicity)

```
# creates a numeric score with range 0-2340
# indicates the total number of discrimination experiences in a year
# higher scores indicate more frequent perceived experience of unfair treatment

# reason is an optional argument
# can limit to participants who provided a particular reason for discrimination, e.g. race or age
# see survey for options

import pandas as pd

def calc_edd_chronicity(survey_df):
    if survey_df is None or survey_df.empty:
        return pd.DataFrame()

    question_ids = [40192380, 40192395, 40192416, 40192451, 40192466,
                    40192489, 40192490, 40192496, 40192519] # 9 specific items
    # 40192380 = In your day-to-day life, how often do people act as if they are afraid of you?
    # 40192395 = In your day-to-day life, how often do people act as if they think you are dishonest?
    # 40192416 = In your day-to-day life, how often do you receive poorer service than other people at restaurants
    #            or stores?
    # 40192451 = In your day-to-day life, how often are you threatened or harassed?
    # 40192466 = In your day-to-day life, how often are you treated with less courtesy than other people?
    # 40192489 = In your day-to-day life, how often are you treated with less respect than other people?
    # 40192490 = In your day-to-day life, how often do people act as if they think you are not smart?
    # 40192496 = In your day-to-day life, how often do people act as if they're better than you are?
    # 40192519 = In your day-to-day life, how often are you called names or insulted?

    # map answer_concept_id to value
    score_mapping = {
        40192465: 0,    # Never
        40192464: 0.5,  # Less than once a year
        40192453: 3,    # A few times a year
        40192461: 36,   # A few times a month
        40192391: 104,  # At least once a week
        40192421: 260,  # Almost everyday
        903096: None    # PMI: Skip, exclude from scoring
    }
```

```python
    df_filtered = survey_df[survey_df['question_concept_id'].isin(question_ids)]
    df_filtered['score'] = df_filtered['answer_concept_id'].map(lambda x: score_mapping.get(x))
    df_filtered = df_filtered[df_filtered['score'] != 999] # remove skips

    # group by person_id and calculate sum score
    df_edd_chronicity = df_filtered.groupby('person_id')['score'].agg(['sum', 'count']).reset_index()
    df_edd_chronicity.columns = ['person_id', 'edd_chronicity', 'answered_questions']
    df_edd_chronicity = df_edd_chronicity[df_edd_chronicity['answered_questions'] == len(question_ids)]
                                                        # include only participants who answered all 9 questions

    # include participants without scores as NA
    df_final = pd.merge(survey_df[['person_id']].drop_duplicates(), df_edd_chronicity[['person_id', 'edd_chronicity']],
                                                              on='person_id', how='left')
    df_final['edd_chronicity'] = df_final['edd_chronicity'].round(2)  # round to 2 decimals

    return df_final

df_edd_chronicity = calc_edd_chronicity(survey_df)
```

Perceived Discrimination in Health Care Settings

Never/Ever Scoring (df_hcd_ever)

```
# creates a binary categorical variable with value TRUE or FALSE
# TRUE denotes that the participant has endorsed perceived discrimination in health care ever

import pandas as pd

def calc_hcd_ever(surve_df):
    question_ids = [40192383, 40192394, 40192423, 40192425, 40192497, 40192503, 40192505] # 7 specific items
    df_filtered = survey_df[surve_df['question_concept_id'].isin(question_ids)]
    # 40192383 = How often does a doctor or nurse act as if he or she is better than you when you go to a doctor's office
    #            or other health care provider?
    # 40192394 = How often do you feel like a doctor or nurse is not listening to what you were saying. when you go to a
    #            doctor's office or other health care provider?
    # 40192423 = How often does a doctor or nurse act as if he or she is afraid of you when you go to a doctor's office or
    #            other health care provider?
    # 40192425 = How often are you treated with less respect than other people when you go to a doctor's office or other
    #            health care provider?
    # 40192497 = How often are you treated with less courtesy than other people when you go to a doctor's office or other
    #            health care provider?
    # 40192503 = How often do you receive poorer service than others when you go to a doctor's office or other health care
    #            provider?
    # 40192505 = How often does a doctor or nurse act as if he or she thinks you are not smart when you go to a doctor's
    #            office or other health care provider?

    # map answer_concept_id to value
    score_mapping = {
        40192465: 0,  # Code for 'Never'
        903096: 999   # Code for 'PMI: Skip'
    }
    df_filtered['score'] = df_filtered['answer_concept_id'].map(lambda x: score_mapping.get(x, 1))
    df_filtered = df_filtered[df_filtered['score'] != 999] # remove skips

    # group by person_id and calculate score
    df_hcd_ever = df_filtered.groupby('person_id')['score'].agg(
        hcd_ever=lambda x: int(1 in x.values),  # Convert boolean to integer
        nrows='count').reset_index()
```

```
    df_hcd_ever = df_hcd_ever[(df_hcd_ever['nrows'] == len(question_ids)) | (df_hcd_ever['hcd_ever'] == 1)]
                                            # include only participants who answered all 7 questions
    df_hcd_ever.drop('nrows', axis=1, inplace=True)

    return df_hcd_ever

df_hcd_ever = calc_hcd_ever(survey_df)
```

Count Scoring (df_hcd_count)

```python
# creates a numeric score with range 0-7
# indicates how many items for which the participant endorsed perceived discrimination in health care
# higher scores indicate greater perceived discrimination in health care

import pandas as pd

def calc_hcd_count(survey_df):
    if survey_df is None or survey_df.empty:
        return pd.DataFrame()

    question_ids = [40192383, 40192394, 40192423, 40192425, 40192497, 40192503, 40192505] # 7 specific items
    # 40192383 = How often does a doctor or nurse act as if he or she is better than you when you go to a doctor's office
    #            or other health care provider?
    # 40192394 = How often do you feel like a doctor or nurse is not listening to what you were saying. when you go to a
    #            doctor's office or other health care provider?
    # 40192423 = How often does a doctor or nurse act as if he or she is afraid of you when you go to a doctor's office or
    #            other health care provider?
    # 40192425 = How often are you treated with less respect than other people when you go to a doctor's office or other
    #            health care provider?
    # 40192497 = How often are you treated with less courtesy than other people when you go to a doctor's office or other
    #            health care provider?
    # 40192503 = How often do you receive poorer service than others when you go to a doctor's office or other health care
    #            provider?
    # 40192505 = How often does a doctor or nurse act as if he or she thinks you are not smart when you go to a doctor's
    #            office or other health care provider?

    # Filter DataFrame for specific question IDs related to healthcare discrimination
    df_filtered = survey_df[survey_df['question_concept_id'].isin(question_ids)]

    # Apply scoring: 0 for 'Never', 1 for any other response, except for 'PMI: Skip' which is marked as 999 for exclusion
    df_filtered['value'] = df_filtered['answer_concept_id'].apply(
        lambda x: 0 if x == 40192465 else (999 if x == 903096 else 1)
    )
    df_filtered = df_filtered[df_filtered['value'] != 999]  # remove skips

    # group by person_id and calculate sum score
    df_hcd_count = df_filtered.groupby('person_id')['value'].agg(['sum', 'count']).reset_index()
```

```python
    df_hcd_count.columns = ['person_id', 'hcd_count', 'answered_questions']

    df_hcd_count = df_hcd_count[df_hcd_count['answered_questions'] == len(question_ids)]
                                         # include only participants who answered all 7 questions
    df_final = df_hcd_count[['person_id', 'hcd_count']]

    # include participants without scores as NA
    df_final = pd.merge(survey_df[['person_id']].drop_duplicates(), df_final, on='person_id', how='left')
    df_final['hcd_count'] = df_final['hcd_count'].round(2)  # round to 2 decimals

    return df_final

df_hcd_count = calc_hcd_count(survey_df)
```

Continuous Scoring: Sum of Items (df_hcd_sum)

```python
# creates a numeric score with range 7-35
# sum of individual item scores
# higher scores indicate greater perceived discrimination in health care

import pandas as pd

def calc_hcd_sum(survey_df):
    question_ids = [40192383, 40192394, 40192423, 40192425, 40192497, 40192503, 40192505] # 7 specific items
    df_filtered = survey_df[survey_df['question_concept_id'].isin(question_ids)]
    # 40192383 = How often does a doctor or nurse act as if he or she is better than you when you go to a doctor's office
    #            or other health care provider?
    # 40192394 = How often do you feel like a doctor or nurse is not listening to what you were saying. when you go to a
    #            doctor's office or other health care provider?
    # 40192423 = How often does a doctor or nurse act as if he or she is afraid of you when you go to a doctor's office or
    #            other health care provider?
    # 40192425 = How often are you treated with less respect than other people when you go to a doctor's office or other
    #            health care provider?
    # 40192497 = How often are you treated with less courtesy than other people when you go to a doctor's office or other
    #            health care provider?
    # 40192503 = How often do you receive poorer service than others when you go to a doctor's office or other health care
    #            provider?
    # 40192505 = How often does a doctor or nurse act as if he or she thinks you are not smart when you go to a doctor's
    #            office or other health care provider?

    # map answer_concept_id to value
    def map_to_score(answer):
        scores = {
            'Never': 1,
            'Rarely': 2,
            'Sometimes': 3,
            'Most of the time': 4,
            'Always': 5,
            'PMI: Skip': 999
        }
        return scores.get(answer, 999)
```

```
    df_filtered['score'] = df_filtered['answer'].apply(map_to_score)
    df_filtered = df_filtered[df_filtered['score'] != 999] # remove skips

    # group by person_id and calculate sum score
    df_hcd_sum = df_filtered.groupby('person_id').agg( hcd_sum=('score', 'sum'),nrows=('score', 'count')).reset_index()
    df_hcd_sum = df_hcd_sum[df_hcd_sum['nrows'] == len(question_ids)]
                                   # include only participants who answered all 7 questions
    df_final = df_hcd_sum[['person_id', 'hcd_sum']]

    # include participants without scores as NA
    df_final = pd.merge(survey_df[['person_id']].drop_duplicates(), df_final, on='person_id', how='left')
    df_final['hcd_sum'] = df_final['hcd_sum'].round(2)  # round to 2 decimals

    return df_final

df_hcd_sum = calc_hcd_sum(survey_df)
```

Continuous Scoring: Item Average (df_hcd_mean)

```python
# creates a numeric score with range 1-5
# mean of individual item scores
# higher scores indicate greater perceived discrimination in health care

import pandas as pd

def calc_hcd_mean(survey_df):
    question_ids = [40192383, 40192394, 40192423, 40192425, 40192497, 40192503, 40192505] # 7 specific items
    df_filtered = survey_df[survey_df['question_concept_id'].isin(question_ids)]
    # 40192383 = How often does a doctor or nurse act as if he or she is better than you when you go to a doctor's office
    #            or other health care provider?
    # 40192394 = How often do you feel like a doctor or nurse is not listening to what you were saying. when you go to a
    #            doctor's office or other health care provider?
    # 40192423 = How often does a doctor or nurse act as if he or she is afraid of you when you go to a doctor's office or
    #            other health care provider?
    # 40192425 = How often are you treated with less respect than other people when you go to a doctor's office or other
    #            health care provider?
    # 40192497 = How often are you treated with less courtesy than other people when you go to a doctor's office or other
    #            health care provider?
    # 40192503 = How often do you receive poorer service than others when you go to a doctor's office or other health care
    #            provider?
    # 40192505 = How often does a doctor or nurse act as if he or she thinks you are not smart when you go to a doctor's
    #            office or other health care provider?

    # map answer_concept_id to value
    score_mapping = {
        'Never': 1,
        'Rarely': 2,
        'Sometimes': 3,
        'Most of the time': 4,
        'Always': 5,
        'PMI: Skip': 999
    }

    df_filtered['score'] = df_filtered['answer'].map(score_mapping)
    df_filtered = df_filtered[df_filtered['score'] != 999] # remove skips
```

```python
    # group by person_id and calculate mean score
    df_hcd_mean = df_filtered.groupby('person_id')['score'].agg(hcd_mean='mean',  nrows='count').reset_index()
    df_hcd_mean = df_hcd_mean[df_hcd_mean['nrows'] == len(question_ids)]
                                        # include only participants who answered all 7 questions
    df_final = df_hcd_mean[['person_id', 'hcd_mean']]

    # include participants without scores as NA
    df_final = pd.merge(survey_df[['person_id']].drop_duplicates(), df_final, on='person_id', how='left')
    df_final['hcd_mean'] = df_final['hcd_mean'].round(2)  # round to 2 decimals

    return df_final

df_hcd_mean = calc_hcd_mean(survey_df)
```

Food Insecurity

df_food_insecurity

```python
# creates a binary categorical variable with value TRUE or FALSE
# TRUE denotes that the participant is at risk or currently experiencing food insecurity

import pandas as pd

def calc_food_insecurity(survey_df):
    question_ids = [40192426, 40192517] # 2 specific items
    df_filtered = survey_df[survey_df['question_concept_id'].isin(question_ids)]
    # 40192426 = Within the past 12 months, were you worried whether the food you had bought just didn't last and you
    #            didn't have money to get more?
    # 40192517 = Within the past 12 months, were you worried whether your food would run out before you got money to
    #            buy more?

    # map answer_concept_id to value
    df_filtered['answer'] = df_filtered['answer'].replace('PMI: Skip', pd.NA) # remove skips
    df_filtered = df_filtered.dropna(subset=['answer'])

    # group by person_id and calculate score
    df_filtered['food_insecurity'] = df_filtered.groupby('person_id')['answer'].transform(
        lambda x: ('Often true' in x.values) or ('Sometimes true' in x.values)
    ).astype(int)  # Convert boolean to integer (True=1, False=0)

    # Count the number of questions answered by each person
    df_filtered['nrows'] = df_filtered.groupby('person_id')['answer'].transform('count')
    df_filtered = df_filtered[df_filtered['nrows'] == len(question_ids)]
                                      # include only participants who answered all 2 questions

    df_food_insecurity = df_filtered[['person_id', 'food_insecurity']].drop_duplicates()

    return df_food_insecurity

df_food_insecurity = calc_food_insecurity(survey_df)
```

Housing Insecurity / Instability

Housing Insecurity (df_housing_insecurity)

```
# creates a binary categorical variable with value TRUE or FALSE
# TRUE denotes that the participant is at risk or currently experiencing housing insecurity
#    (i.e., moved 2 or more times in the past year)

import pandas as pd

def calc_housing_insecurity(survey_df):
    if survey_df is not None:
        question_id = 40192441  # 1 specific item
        df_filtered = survey_df[survey_df['question_concept_id'] == question_id]
        # 40192441 = In the last 12 months, how many times have you or your family moved from one home to another?
        #            Number of moves in past 12 months:

        # map answer_concept_id to value
        df_filtered= df_filtered[df_filtered['answer'] != 'PMI: Skip'] # remove skips
        df_filtered['housing_insecurity'] = df_filtered['answer'].apply(
            lambda x: False if x in ['0', '1'] else True
        )
        df_housing_insecurity = df_filtered[['person_id', 'housing_insecurity']]

        # include participants without scores as NA
        df_housing_insecurity = pd.merge(df_housing_insecurity, survey_df.groupby('person_id').size().reset_index(),
                                                                how='right', on='person_id')

        return df_housing_insecurity

df_housing_insecurity = calc_housing_insecurity(survey_df)
```

Number of Moves (df_num_moves)

```python
# creates a numeric variable representing the number of moves in the past year

import pandas as pd

def calc_num_moves(survey_df):
    if survey_df is not None:
        question_id = 40192441  # 1 specific item
        df_filtered = survey_df[survey_df['question_concept_id'] == question_id]
        # 40192441 = In the last 12 months, how many times have you or your family moved from one home to another?
        #            Number of moves in past 12 months:

        # map answer_concept_id to value
        df_filtered = df_filtered[df_filtered['answer'] != 'PMI: Skip'] # remove skips
        df_filtered['num_moves'] = pd.to_numeric(df_filtered['answer'], errors='coerce')
        df_num_moves = df_filtered[['person_id', 'num_moves']]

        # include participants without scores as NA
        df_num_moves = pd.merge(df_num_moves, survey_df.groupby('person_id').size().reset_index(), how='right',
                                                                                              on='person_id')

        return df_num_moves

df_num_moves = calc_num_moves(survey_df)
```

Housing Quality

df_housing_quality

```python
# creates a binary categorical variable with value TRUE or FALSE
# TRUE denotes that the participant is endorsing a housing need, (i.e., selected at least one problem)

import pandas as pd

def calc_housing_quality(survey_df):
    if survey_df is not None:
        question_id = 40192402  # 1 specific item
        df_filtered = survey_df[survey_df['question_concept_id'] == question_id]
        # 40192402 = Think about the place you live. Do you have problems with any of the following? Select all that apply.

        # map answer_concept_id to value
        answer_mapping = {
            'None of the above': 0,
            'PMI: Skip': 999
        }
        df_filtered['score'] = df_filtered['answer'].map(answer_mapping).fillna(1)
        df_filtered = df_filtered[df_filtered['score'] != 999] # remove skips

        # group by person_id and calculate score
        df_housing_quality = df_filtered.groupby('person_id')['score'].apply(
          lambda x: 1 if x.any() else 0).reset_index()
        df_housing_quality.rename(columns={'score': 'housing_quality'}, inplace=True)

        return df_housing_quality

df_housing_quality = calc_housing_quality(survey_df)
```

Perceived Stress

Sum Scoring (df_stress_sum)

```python
# creates a numeric score with range 0-40
# sum of individual item scores
# higher scores indicate higher levels of perceived stress

import pandas as pd

def calc_stress_sum(survey_df):
    question_ids = [40192381, 40192396, 40192419, 40192445, 40192449, 40192452,
                    40192462, 40192491, 40192506, 40192525] # 10 specific items
    df_filtered = survey_df[survey_df['question_concept_id'].isin(question_ids)]
    # 40192381 = In the last month, how often have you felt that you were unable to control the important things in your
    #            life?
    # 40192396 = In the last month, how often have you been angered because of things that were outside of your control?
    # 40192419 = In the last month, how often have you felt confident about your ability to handle your personal problems?
    # 40192445 = In the last month, how often have you felt that you were on top of things?
    # 40192449 = In the last month, how often have you been able to control irritations in your life?
    # 40192452 = In the last month, how often have you been upset because of something that happened unexpectedly?
    # 40192462 = In the last month, how often have you felt difficulties were piling up so high that you could not overcome
    #            them?
    # 40192491 = In the last month, how often have you felt nervous and "stressed"?
    # 40192506 = In the last month, how often have you found that you could not cope with all the things that you had to do?
    # 40192525 = In the last month, how often have you felt that things were going your way?

    # map answer_concept_id to value
    scoring_mapping = {
        'Never': 0,
        'Almost Never': 1,
        'Sometimes': 2,
        'Fairly Often': 3,
        'Very Often': 4,
        'PMI: Skip': 999
    }
    df_filtered['score'] = df_filtered['answer'].map(scoring_mapping)
    df_filtered = df_filtered[df_filtered['score'] != 999]  # remove skips
```

```python
    # group by person_id and calculate sum score
    df_filtered['answered_questions'] = df_filtered.groupby('person_id')['question_concept_id'].transform('nunique')
    df_filtered = df_filtered[df_filtered['answered_questions'] == len(question_ids)]
                                      # include only participants who answered all 10 questions

    reverse_scoring_question_ids = [40192419, 40192445, 40192449, 40192525]  # reverse scoring
    df_filtered.loc[df_filtered['question_concept_id'].isin(reverse_scoring_question_ids),'score'] = 4-df_filtered['score']

    df_stress_sum = df_filtered.groupby('person_id')['score'].sum().reset_index(name='stress_sum')

    return df_stress_sum

df_stress_sum = calc_stress_sum(survey_df)
```

Categorical Scoring (df_stress_category)

```
# creates an ordinal categorical variable with values 'Low', 'Moderate', 'High'
# 'Low' denotes perceived stress score 0-13
# 'Moderate' denotes perceived stress score 14-26
# 'High' denotes perceived stress score 27-40

import pandas as pd

def calc_stress_category_and_sum(survey_df):
    question_ids = [40192381, 40192396, 40192419, 40192445, 40192449, 40192452,
                    40192462, 40192491, 40192506, 40192525] # 10 specific items
    df_filtered = survey_df[survey_df['question_concept_id'].isin(question_ids)]
    # 40192381 = In the last month, how often have you felt that you were unable to control the important things in your
    #            life?
    # 40192396 = In the last month, how often have you been angered because of things that were outside of your control?
    # 40192419 = In the last month, how often have you felt confident about your ability to handle your personal problems?
    # 40192445 = In the last month, how often have you felt that you were on top of things?
    # 40192449 = In the last month, how often have you been able to control irritations in your life?
    # 40192452 = In the last month, how often have you been upset because of something that happened unexpectedly?
    # 40192462 = In the last month, how often have you felt difficulties were piling up so high that you could not overcome
    #            them?
    # 40192491 = In the last month, how often have you felt nervous and "stressed"?
    # 40192506 = In the last month, how often have you found that you could not cope with all the things that you had to do?
    # 40192525 = In the last month, how often have you felt that things were going your way?

    # map answer_concept_id to value
    scoring_mapping = {
        'Never': 0,
        'Almost Never': 1,
        'Sometimes': 2,
        'Fairly Often': 3,
        'Very Often': 4,
        'PMI: Skip': None  # Changed from 999 to None for easier exclusion of skipped questions
    }

    df_filtered['score'] = df_filtered['answer'].map(scoring_mapping)
    df_filtered.dropna(subset=['score'], inplace=True) # remove skips
```

```python
    # group by person_id and calculate sum score
    df_filtered['answered_questions'] = df_filtered.groupby('person_id')['question_concept_id'].transform('nunique')
    df_filtered = df_filtered[df_filtered['answered_questions'] == len(question_ids)]
                                          # include only participants who answered all 10 questions

    reverse_scoring_question_ids = [40192419, 40192445, 40192449, 40192525] #  reverse scoring
    df_filtered.loc[df_filtered['question_concept_id'].isin(reverse_scoring_question_ids), 'score'] = 4-df_filtered['score']

    df_stress = df_filtered.groupby('person_id')['score'].sum().reset_index(name='stress_sum')

    # Categorize stress levels based on the stress sum
    def categorize_stress(score):
        if score <= 13:
            return 'Low'
        elif score <= 26:
            return 'Moderate'
        else:
            return 'High'

    df_stress['stress_category'] = df_stress['stress_sum'].apply(categorize_stress)

    return df_stress

df_stress_category = calc_stress_category_and_sum(survey_df)
```

Daily Spiritual Experiences

df_spirit

```python
# creates a numeric score with range 6-36
# sum of individual item scores
# higher scores indicate more daily religious or spiritual experiences

import pandas as pd

def calc_spiritual_experience_score(survey_df):
    question_ids = [40192401, 40192415, 40192443, 40192471, 40192475, 40192498] # 6 specific items
    # 40192401 = How often do you feel deep inner peace or harmony?
    # 40192415 = How often do you feel that you are spiritually touched by the beauty of creation?
    # 40192443 = How often do you desire to be closer to or in union with God (or a higher power)?
    # 40192471 = How often do you feel God's (or a higher power's) love for you, directly or through others?
    # 40192475 = How often do you find strength and comfort in your religion?
    # 40192498 = How often do you feel God's (or a higher power's) presence?

    # map answer_concept_id to value
    score_mapping = {
        'I do not believe in God (or a higher power)': 1,
        'I am not religious': 1,
        'Never or almost never': 1,
        'Once in a while': 2,
        'Some days': 3,
        'Most days': 4,
        'Every day': 5,
        'Many times a day': 6
    }

    df_filtered = survey_df[
        survey_df['question_concept_id'].isin(question_ids) &
        (survey_df['answer'] != 'PMI: Skip') &   # remove skips
        (~survey_df['answer'].isna())
    ]

    df_filtered['score'] = df_filtered['answer'].map(score_mapping)
```

```
    # group by person_id and calculate sum score
    df_spirit = df_filtered.groupby('person_id').agg(
        spirit=('score', 'sum'),
        nrows=('score', 'count')
    )

    df_spirit = df_spirit[df_spirit['nrows'] == len(question_ids)].reset_index()[['person_id', 'spirit']]
                                     # include only participants who answered all 6 questions

    return df_spirit

df_spirit = calc_spiritual_experience_score(survey_df)
```

Religious Service Attendance

df_religious_attendance

```
# creates an ordinal categorical indicating the frequency of attending religious meetings or services

import pandas as pd

def calc_religious_attendance(survey_df):
    if survey_df is not None:
        question_id = 40192470 # 1 specific item
        df_filtered = survey_df[survey_df['question_concept_id'] == question_id]
        # 40192470 = How often do you go to religious meetings or services?

        # map answer_concept_id to value
        df_filtered = df_filtered[df_filtered['answer'] != "PMI: Skip"] # remove skips
        df_filtered.dropna(subset=['answer'], inplace=True)
        df_filtered['religious_attendance'] = df_filtered['answer']

        result_df = df_filtered[['person_id', 'religious_attendance']].drop_duplicates()
        summary_df = survey_df[['person_id']].drop_duplicates()

        # include participants without scores as NA
        df_religious_attendance = pd.merge(summary_df, result_df, on='person_id', how='left')

        return df_religious_attendance

df_religious_attendance = calc_religious_attendance(survey_df)
```

English Proficiency

Speaks Other Language at Home (df_other_language)

```
# creates a nominal categorical variable with values 'Yes', 'No', or 'PMI: Prefer Not To Answer'
# 'Yes' denotes that the participant speaks a language other than English at home

import pandas as pd

def calc_other_language(survey_df):
    if survey_df is not None:
        df_filtered = survey_df[survey_df['question_concept_id'] == 40192526] # 1 specific item
        # 40192526 = Do you speak a language other than English at home?

        # map answer_concept_id to value
        df_filtered = df_filtered[df_filtered['answer'] != "PMI: Skip"] # remove skips
        df_filtered['other_language'] = df_filtered['answer']

        result_df = df_filtered[['person_id', 'other_language']].drop_duplicates()
        summary_df = survey_df[['person_id']].drop_duplicates()

        # include participants without scores as NA
        df_other_language = pd.merge(summary_df, result_df, on='person_id', how='left')

        return df_other_language

df_other_language = calc_other_language(survey_df)
```

Level of English Proficiency (df_english_level)

```
# creates an ordinal categorical variable describing level of proficiency in English for participants who
#   endorsed speaking a language other than English at home

import pandas as pd

def calc_english_level(survey_df):
    if survey_df is not None:
        df_filtered = survey_df[survey_df['question_concept_id'] == 40192529] # 1 specific item
        # 40192529 = Since you speak a language other than English at home, we are interested in your own thoughts about
        #            how well you think you speak English. Would you say you speak English...

        # map answer_concept_id to value
        df_filtered = df_filtered[df_filtered['answer'] != "PMI: Skip"] # remove skips
        df_filtered['english_level'] = df_filtered['answer']

        result_df = df_filtered[['person_id', 'english_level']].drop_duplicates()
        summary_df = survey_df[['person_id']].drop_duplicates()

        # include participants without scores as NA
        df_english_level = pd.merge(summary_df, result_df, on='person_id', how='left')

        return df_english_level

df_english_level = calc_english_level(survey_df)
```

English Proficient (df_english_proficient)

```
# creates a nominal categorical variable with values 'Proficient', 'Not proficient', or 'Unknown' for participants who
#   endorsed speaking a language other than English at home
# 'Proficient' denotes participants who endorsed speaking English 'Very well' or 'Well'
# 'Not proficient' denotes participants who endorsed speaking English 'Not well' or 'Not at all'

import pandas as pd

def calc_english_proficient(survey_df):
    if survey_df is not None:
        df_filtered = survey_df[survey_df['question_concept_id'] == 40192529] # 1 specific item
        # 40192529 = Since you speak a language other than English at home, we are interested in your own thoughts about
        #            how well you think you speak English. Would you say you speak English...

        # map answer_concept_id to value
        proficiency_mapping = {
            40192435: "Proficient",  # Very well
            40192510: "Proficient",  # Well
            40192405: "Not proficient",  # Not well
            40192387: "Not proficient",  # Not at all
            903087: "Unknown",  # PMI: Don't Know
            903079: "Unknown"  # PMI: Prefer Not To Answer
        }
        df_filtered['english_proficient'] = df_filtered['answer_concept_id'].map(proficiency_mapping).fillna("none")
        df_filtered = df_filtered[df_filtered['english_proficient'] != "none"] # remove skips

        result_df = df_filtered[['person_id', 'english_proficient']].drop_duplicates()
        summary_df = survey_df[['person_id']].drop_duplicates()

        # include participants without scores as NA
        df_english_proficient = pd.merge(summary_df, result_df, on='person_id', how='left')

        return df_english_proficient

df_english_proficient = calc_english_proficient(survey_df)
```
